# Bioherbicidal Activity and Metabolic Profiling of Potent Allelopathic Plant Fractions Against Major Weeds of Wheat—Way Forward to Lower the Risk of Synthetic Herbicides

**DOI:** 10.3389/fpls.2021.632390

**Published:** 2021-09-10

**Authors:** Sobia Anwar, Saadia Naseem, Saira Karimi, Muhammad Rafique Asi, Ahmed Akrem, Zahid Ali

**Affiliations:** ^1^Plant Biotechnology and Molecular Pharming Laboratory, Department of Biosciences, COMSATS University Islamabad (CUI), Islamabad, Pakistan; ^2^Nuclear Institute for Agriculture and Biology (NIAB), Faisalabad, Pakistan; ^3^Department of Botany, Institute of Pure and Applied Biology, Bahauddin Zakariya University, Multan, Pakistan

**Keywords:** plant extracts, allelopathy, bio-herbicide, phenolic compounds, weed management

## Abstract

The productivity of major field crops is highly compromised due to weed infestation. Inefficient weed management practices and undue and excessive use of chemical herbicides have drastically contaminated the environment and human health, in addition to resistance development in weed species. Therefore, utilization of allelopathic plants to explore phytochemicals as potent organic alternatives to such chemical herbicides has become indispensable. The current study evaluates the comparative bio-herbicidal potential of methanolic extracts of castor (*Ricinus communis*), artemisia (*Artemisia santolinifolia*), wheat (*Triticum aestivum*), and sorghum (*Sorghum bicolor*) to suppress growth of major weeds, i.e., wild mustard (*Sinapis arvensis*), Italian ryegrass (*Lolium multiflorum*), and carrot grass (*Parthenium hysterophorus*). The results demonstrated a concentration-dependent effect on weeds’ growth. Overall, *in vitro* seed germination was reduced from 60 to 100% in response to 5% (w/v) extract concentration. Significant reduction in radicle length, hypocotyl length, and fresh biomass of the weeds was also observed. A strong inhibitory effect was seen in *in vivo* pot experiments, revealing that application of 10–20% methanolic extracts induced permanent wilting and substantial reduction in the chlorophyll content of weeds along with 20–80% increase in oxidative stress. Artemisia showed the most significant allelopathic effect, on account of highest phenolic and flavonoid contents, followed by castor, wheat, and sorghum, against *S. arvensis*, *L. multiflorum*, and *P. hysterophorus*, respectively. Phytochemical analysis, through high-performance liquid chromatography (HPLC), also exhibited a correlation between extract’s phytotoxicity and their antioxidant potential due to their major constituents (rutin, quercetin, catechin, gallic acid, vanillic acid, syringic acid, ferulic acid, *p*-hydroxy benzoic acid, *p*-coumaric acid, and sinapic acid), among the total of 13 identified in methanolic fractions. Comprehensive profiling of allelochemicals with liquid chromatography–mass spectrometry (LC-MS) determined 120, 113, 90, and 50 derivates of phenolic acids, flavonoids, and alkaloids, reported for the first time through this study, demonstrating significant allelopathic potential of the targeted plant fractions, which can be explored further to develop a sustainable bio-herbicidal formulation.

## Introduction

Weed infestation contributes to annual losses of up to $43 billion in the United States and Canada only (Wychen, 2016), while in Pakistan, these losses count up to US$ 0.93–1.62 billion (Cheema and Khaliq, 2002). Therefore, agricultural productivity is severely compromised due to the weed’s influx, causing 60–70% yield reduction in wheat (*Triticum aestivum* L.) (Siddiqui et al., 2010). Major weed species causing such destructive losses to major crops in different regions include carrot grass (*Parthenium hysterophorus*), canary grass (*Phalaris minor*), wild mustard (*Sinapis arvensis*), wild spinach (*Chenopodium album*), milk thistle (*Silybum marianum*), nut grass (*Cyperus rotundus* L.), small flowered nut sedge (*Cyperus difformis* L.), jungle rice (*Echinochloa colona* L.), burclover (*Medicago denticulata*), toothed dock (*Rumex dentatus*), wild oat (*Avena fatua*), and Italian ryegrass (*Lolium* spp.) (Tanveer et al., 2003; Khan et al., 2007; Shabbir and Bajwa, 2007; Rajput et al., 2008; Sarwar et al., 2013; Iqbal et al., 2017; Tahira and Khan, 2017). Among these, *S. arvensis* is specifically known to prevail abundantly in wheat (Gherekhloo et al., 2018) with a density of approximately 40%, reducing ∼43% grain yield (Dhima and Eleftherohorinos, 2005; Behdarvand et al., 2013; Erman et al., 2020). *Lolium multiflorum* is also a great competitor (Appleby and Brewster, 1992), being 46% prevalent in cultivated wheat area (Scursoni et al., 2012), whereby reducing its production up to 90% (Hashem et al., 1998; Bailey and Wilson, 2003; Worthington et al., 2013; Andreasen et al., 2020). *P. hysterophorus* is another emerging concern that is adversely hindering wheat growth (Khan et al., 2012; Bajwa et al., 2016) in addition to inducing dermatitis, asthma, bronchitis, and many other ailments in the human population (Nadeem et al., 2005; Chandra Roy and Munan Shaik, 2013; Lalita, 2018). Conventional weed management strategies restrict weed growth for a short duration after which mostly weeds regrow in the field (Farooq et al., 2020), whereas extensive use of chemical herbicides has significant negative impacts on the environment, due to residual toxicity to human health, soil, and groundwater (Marwat et al., 2011). This disturbed the soil micro-ecosystem by affecting non-target microbiota and most importantly the development of herbicide resistance in major weed species (Hussain et al., 2010). Presently, about 500 weed species have been reported that have developed resistance against 166 herbicides belonging to more than 25 known modes of actions worldwide (Heap, 2018). *S. arvensis*, *L. multiflorum*, and *P. hysterophorus* are among those weeds developing resistance to photosystem II inhibitor, fatty acid inhibitor, synthetic auxin inhibitor, acetolactate synthase (ALS), acetyl CoA carboxylase (ACCase), and 5-enolpyruvylshikimate-3-phosphate synthase (EPSPS), barring herbicides worldwide (Topuz et al., 2015; Heap, 2018; Khaledi et al., 2019). Considering these negative impacts of conventional weed control methods and increasing incidents of herbicide resistance, continued research on potential allelopathic plants, as an organic alternative, is essential for safer weed management practices. Naturally derived phytotoxic extracts may be implemented for agricultural sustainability (Soltys et al., 2013). Various plants, including wheat, rice, pea, white mustard, barley, and sunflower, have been investigated to determine their allelopathicity against weeds (Wu et al., 2001; Kato-Noguchi, 2003; Smith and Dilday, 2003; Alcántara et al., 2011; Arif et al., 2015; Christenson et al., 2015). In this study, *Artemisia santolinifolia*, *Ricinus communis*, *T. aestivum*, and *Sorghum bicolor* were selected as donor plants for allelopathic investigation against weeds (*S. arvensis*, *L. multiflorum*, and *P. hysterophorus*). Therapeutic and nutritional importance of mentioned plants is substantially evident from earlier studies. These plants have anti-carcinogenic, anti-inflammatory, anti-diabetic, and hepatoprotective activities, as they are strong antioxidants owing to the presence of phenolic acids, flavonoids, alkaloids, and fatty acids in them (Melguizo-Melguizo et al., 2014; Žilić, 2016; Kumar, 2017; Rao et al., 2018). High antioxidant potential of plants could be a source of their allelopathicity as well (El-Gawad et al., 2019). Aqueous extracts of *R. communis*, *T. aestivum*, and *S. bicolor* have also been employed for weed suppression (Wu et al., 2001; Mahmood et al., 2013; Naz and Bano, 2014; Saadaoui et al., 2015; Renathielly et al., 2016; Al-Samarai et al., 2018; Eassa et al., 2018; Storozhyk et al., 2019), but their methanolic extracts have not been investigated as yet. Water extracts of different species of artemisia were also used as a bio-control agent against weeds (Lydon et al., 1997; Barney et al., 2005; Onen, 2013; Kapoor et al., 2019; Benarab et al., 2020); but allelopathy of *A. santolinifolia* is still to be established. Moreover, the detailed allelochemical profiling of these species is also lacking.

Considering the increased density of weeds among major crops including wheat, there is a consistent need to explore new allelopathic plants and their phytotoxins. For this purpose, screening of plants should be done through easy and rapid bioassays that may efficiently identify the active allelochemicals through chromatographic techniques (Duke, 2015). Allelochemicals can prove to be an eco-friendly and equally efficient weed control approach with minimum environmental hazards due to their structural organizations, short half-life in soils, and synergistic action when released into the environment (Soltys et al., 2013). Diverse classes of allelochemicals, including phenols, terpenes, flavonoids, fatty acids, and steroids, are known to have phytotoxicity against multiple weeds by inhibiting their photosynthetic pathways, disrupting activity of metabolic enzymes, adversely affecting mitochondrial respiration, inhibiting mitotic process, increasing oxidative stress, altering lignin composition, inhibiting cell division and enzyme functions, and inactivating protein synthesis (Li et al., 2010; Golisz et al., 2011; Borella et al., 2014; Wang et al., 2014). Contrary to synthetic herbicides, these natural compounds act by blocking biochemical pathways of weeds in a natural way and, hence, may be considered as a sustainable alternative to chemical herbicides (Dayan et al., 1999). Thus, the present study has been designed to investigate herbicidal potential of *R. communis*, *A. santolinifolia*, *T. aestivum*, and *S. bicolor* extracts against major weeds like *S. arvensis*, *L. multiflorum*, and *P. hysterophorus* and to identify and characterize potent allelochemicals in them. Characterization of novel allelopathins in such plants may assist in providing a basic platform for organically developed herbicide formulation to manage weed infestation in wheat.

## Materials and Methods

For allelopathic potential investigations of castor, artemisia, wheat, and sorghum, plants were selected based on their antioxidant potential (Melguizo-Melguizo et al., 2014; Terpinc et al., 2016; Santos et al., 2018; Murimwa et al., 2019). Test weeds included *Sinapis arvensis*, *Lolium multiflorum*, and *Parthenium hysterophorus*. These species were selected based on their prevalence in crops and their herbicide-resistance development (Scursoni et al., 2012; Bajwa et al., 2016; Erman et al., 2020).

### Plant Extract Preparation

Wheat and sorghum accessions (36003 and 9978, respectively) were obtained from National Agricultural Research Centre (NARC), Islamabad, Pakistan. These accessions were grown under glass house conditions and were harvested after 1 month of seed germination. Artemisia plants were collected in July before flowering stage, from Khunjerab National Park (KNP), Pakistan, while castor plants were collected from the local area of Islamabad. For extraction of phenolic compounds, five whole plants of each species were separately washed and shade dried before maceration with liquid nitrogen. Methanol was used as solvent, as it prevents phytochemical degradation by oxidative enzymes, i.e., polyphenol oxidase and peroxidase (POD), which are active in aqueous solutions (Duke, 2015). Macerated plant material (20 g) was soaked in methanol (100 ml) in a conical flask. The flasks were incubated in dark at room temperature for 72 h. Following incubation, the flasks were regularly shaken to allow efficient extraction of phytochemicals. Extracts were filtered through filter paper (pore size 2.5 μm), and the filtrate was subsequently run through filtration assembly (DEKKER, Model #A300, filter pore size 0.45 μm), under gravity, for complete removal of contaminants. Resulting 20% (w/v) castor and artemisia extracts served as stock solution and were stored at –20°C.

Each methanolic extract was then diluted to prepare 1, 3, and 5% solutions for plate bioassay. For soil (pot) experiments, 10, 15, and 20% concentration dilutions were prepared. The same dilutions were prepared with pure solvent, to treat control seeds/seedlings for plate and soil bio-assays, whereas sterile distilled water was used as a positive control in both experiments. The effect of solvent was normalized to aqueous control before data analysis for each experiment.

### Phytotoxicity Studies on *Sinapis arvensis*, *Lolium multiflorum*, and *Parthenium hysterophorus*

#### Seed Germination Bioassay

Seeds of *S. arvensis*, *L. multiflorum*, and *P. hysterophorus* were collected from the Department of Weed Science, Peshawar, Pakistan, and were surface sterilized with 5% sodium hypochlorite (1 min) to inactivate any microbes and washed three times with autoclaved double-distilled water (Ali et al., 2010). The sterilized seeds (6–10) of each weed species were placed separately in glass Petri plates (90 mm × 10 mm) containing autoclaved filter paper (Whatman no. 42) in triplicates. Subsequently, 1 ml of each extracts was applied to evenly moisten filter paper. The control group received the same amount of methanol (1, 3, and 5%) and sterile water. Plates were sealed with parafilm and incubated in a growth room at 24°C and 16/8-h light/dark photoperiod. Each treatment was repeated three times at an interval of 48 h each. Seed germination count of individual weed was recorded from 10 to 15 days after germination to calculate germination index and germination percentage of each, as follows (Scott et al., 1984).


$$\mathrm{GI}=\frac{\mathrm{Number}\mathrm{of}\mathrm{seeds}\mathrm{germinated}}{\mathrm{First}\mathrm{days}\mathrm{of}\mathrm{count}}+\frac{\mathrm{Number}\mathrm{of}\mathrm{seeds}\mathrm{germinated}}{\mathrm{Last}\mathrm{days}\mathrm{of}\mathrm{count}}$$

$$G\%=\frac{\mathrm{Number}\mathrm{of}\mathrm{seeds}\mathrm{germinated}}{\mathrm{Total}\mathrm{number}\mathrm{of}\mathrm{seeds}}\times 100$$

#### *In vitro* Evaluation of Methanolic Fractions on Seedling Growth

The effect of methanolic fractions (1, 3, and 5%) of castor, artemisia, wheat, and sorghum were investigated through plate bioassays. Several surface-sterilized seeds of *S. arvensis*, *L. multiflorum*, and *P. hysterophorus* were allowed to germinate separately on autoclaved filter papers (Whatman no. 42), moistened with 1 ml of sterile water, contained in Petri plates, under the same conditions as described above. After 7 days of germination, 6–10 similarly sized seedlings of *P. hysterophorus*, *S. arvensis*, and *L. multiflorum* were selected for bioassay. Each extract was applied uniformly (1 ml in each plate) to seedlings, while the control group was inoculated with the same amount of sterile water. The solvent control group was given 1, 3, and 5% methanol. Treatments were repeated thrice on alternate days. After 5 days of each experiment, hypocotyl length (mm), radicle length (mm), and fresh biomass (mg) of each seedling were measured.

### Phytochemical Analysis

Total phenolic contents (TPCs) and total flavonoid contents (TFCs) of 1, 3, and 5% of castor (CE), artemisia (AE), wheat (TE), and sorghum (SE) extracts were determined separately by following the method described by Kamtekar et al. (2014).

#### Total Phenolic Acid Content Assay

TPC of extracts was determined by Folin–Ciocalteu (FC) method (Kamtekar et al., 2014). Stock solution of gallic acid (100 μg of gallic acid per ml of methanol) was used to prepare the following dilutions: 50, 25, 12.5, and 6.25 μg ml^–1^. For TPC estimation, 120 μl of gallic acid or each extract fraction was mixed in 600 μl of FC reagent in a 15-ml Falcon tube, followed by incubation for 5 min. Sodium carbonate (7%) was then added in the solution, volume was adjusted up to 10 ml with sterile distilled water, and the solution was allowed to stand in dark at room temperature for 30 min. Absorbance was read at 760-nm wavelength by a spectrophotometer (SPECORD 50). Solution containing only the reagents except the extract or standard was considered as a blank. TPC in samples was estimated by gallic acid calibration curve, and results were expressed as mg of gallic acid equivalent (GAE)/100 g dry mass of plant material (Kamtekar et al., 2014).

#### Total Flavonoid Content Assay

TFC of each extract was determined by using aluminum chloride colorimetric assay (Kamtekar et al., 2014). Quercetin standard solution (1,000 μg of quercetin per ml of methanol) was used to prepare following dilutions: 100, 200, 400, 600, and 800 μg ml^–1^. To estimate TFC, 1 ml of each fraction or quercetin standard was thoroughly mixed in 4 ml of sterile water and 0.3 ml of 5% NaNO_2_ in a 15-ml tube. The mixture was allowed to stand at room temperature for 5 min. Subsequently, 0.3 ml of 10% AlCl_3_ and 2 ml of 1 M NaOH solution were added at the sixth minute. The volume of the mixture was raised up to 10 ml with sterile distilled water. The final solution was used to measure absorbance at 510 nm on spectrophotometer (SPECORD 50) using visible range. Solution containing all the chemicals except the extract/standard was used as a blank. TFC was determined by quercetin calibration curve, and the results were expressed as quercetin equivalent (QE) in mg/100 g dry mass of plant material (Kamtekar et al., 2014).

### *In vivo* Evaluation of Methanolic Fractions on Seedling Growth

To determine the optimized phytotoxic extract concentration for weed growth suppression in soil, multiple seeds were sown in pots (7.5 × 7.8 cm) containing 100 g mixed compost material and incubated in a growth room at 24°C temperature and 16/8-h light/dark photoperiod. After seedling emergence, thinning was done to maintain uniform plant density of 3, 5, and 15 seedlings of *P. hysterophorus*, *L. multiflorum*, and *S. arvensis* per pot, respectively. In the preliminary screening assessment, lower concentrations of extracts did not affect the weed growth. Therefore, 10, 15, and 20% concentrations were applied at 5 ml per pot of each solution to 1-week-old seedlings on alternate days (days 1, 3, and 5). The same amount of 20% methanol and water was applied to the control.

### Chlorophyll Content Assay

Treated weed seedlings (10, 15, and 20% of each extract) were harvested for total chlorophyll content analysis. Leaves were macerated in liquid nitrogen to determine chlorophyll contents. Ground leaves’ material (0.5 g) in 1.5-ml tubes was mixed with 1 ml of dimethyl sulfoxide (DMSO). Chlorophyll was allowed to dissolve into DMSO by incubating the tubes at 60°C temperature for 30 min. Absorbance were recorded in spectrophotometer (SPECORD 50) at 645 and 663 nm for chlorophyll pigments a and b, respectively. Total chlorophyll concentration was estimated according to the formula equation by Barnes et al. (1992).

### Biochemical Analysis of Weeds Post Extract Treatment

Biochemical analysis of weeds on the effect of 20% allelopathic plant extracts (CE, AE, TE, and SE) was determined through enzymatic [catalase (CAT), superoxide dismutase (SOD), POD] and non-enzymatic assays [malondialdehyde (MDA)]. All analyses were performed spectrophotometrically. Crude enzyme extract was prepared separately from leaves of *S. arvensis*, *L. multiflorum*, and *P. hysterophorus* after permanent wilting post-treatments. Fifty milligrams of each sample was macerated with liquid nitrogen followed by homogenization with 50 mM of phosphate buffer (pH 7.0). The homogenate was centrifuged at 4°C for 20 min at 10,000 × g. Supernatant was collected as enzyme extract and subsequently used for antioxidant enzyme assays. The activity of various enzymes in response to allelopathic stress was determined as follows.

#### Catalase Activity

CAT activity was used to determine the rate of decomposition of H_2_O_2,_ measuring spectrophotometrically at room temperature by monitoring the decrease in absorbance at 240 nm as per the method of Aebi (1974), with some modifications. The reaction mixture (2 ml) contained 100 mM of phosphate buffer of pH 7.0, 40 mM of H_2_O_2_, and 0.2 ml of enzyme extract. One unit was defined as the amount of enzyme that caused an absorbance change of 0.1 per minute under assay conditions. The extinction coefficient of CAT enzyme is 39.4 mM min^–1^, and concentration of H_2_O_2_ was expressed as mM of H_2_O_2_ oxidized min^–1^ g^–1^ fresh weight (FW).

#### Superoxide Dismutase Activity

SOD activity was determined by measuring its ability to inhibit the photoreduction of nitroblue tetrazolium chloride (NBT) by following Beauchamp and Fridovich (1971) method with some modifications. The reaction mixture (1 ml) comprised 63 μM of NBT, 13 mM of L-methionine, 0.1 mM of EDTA, 13 mM of riboflavin, 0.05 M of sodium carbonate, 50 mM of potassium phosphate buffer of pH 7.8, and 0.125 ml of enzyme extract. Distilled water (0.125 ml) was used instead of extract in the control. The reaction mixture was placed in a fluorescent lamp for 15 min at 25°C to stimulate the reaction. The reaction was stopped by dark incubation for 15 min. The blue-colored formazan formed by photochemical reduction was measured as increase in absorbance at 560 nm. Appropriate controls devoid of enzyme extract were used as blank controls incubated in the dark. One SOD unit was defined as the amount of enzyme causing 50% of the NBT photoreduction inhibition. The extinction coefficient of enzyme is 26.6 mM min^–1^ and expressed as units of enzyme activity mM min^–1^ g^–1^ FW.

#### Peroxidase Activity

POD activity was analyzed by determining the absorbance at 470 nm caused by oxidation of guaiacol to tetraguaiacol as describe by Mandal et al. (2008), with some modifications. The final reaction mixture (3 ml) contained 20 mM of guaiacol (0.1 ml), 0.1 mM of potassium phosphate buffer (pH 5.0) (2.5 ml), 40 mM of H_2_O_2_ (0.2 ml), and the enzyme extract (0.2 ml). The extinction coefficient of POD enzyme is 26.6 mM cm^–1^. POD unit was taken as the amount of protein required to oxidize 1 mM of tetraguaiacol per minute and calculated as mM min^–1^ g^–1^ FW.

#### Lipid Peroxidase (Malondialdehyde) Activity

The MDA content was determined to test the level of lipid peroxidation as described by Heath and Packer (1968), with some modifications. Fresh leaves of the target weed (0.1 g of each) were homogenized with 1.0% (w/v) trichloroacetic acid (TCA). The homogenate was centrifuged at 10,000 × g for 15 min at 4°C, and 0.8 ml of the supernatant was added to 2 ml 0.5% (v/v) thiobarbituric acid (TBA) in 20% TCA. The reaction mixture was incubated at 95°C for 15 min in water bath. The reaction was stopped by incubating samples on ice for 10 min. Mixture was then centrifuged 10,000 × g for 5 min. The absorbance of supernatant was measured at 532 and 600 nm. The MDA concentration was calculated by using the extinction coefficient of 155 mM cm^–1^. The concentrations were expressed as mM g^–1^ FW.

### Crude Extract Fractionation Through Solid-Phase Extraction

To remove impurities from crude extracts, fractionations were performed individually using solid-phase extraction (SPE) assembly (Thermo Scientific 24-Port Glass Box Vacuum Manifold) (Głowniak et al., 1996). Methanol was utilized for fractionation, as most phenolic compounds are soluble in it. Initially, the SPE cartridge of 3 ml (CMWBOND, 500 mg) was conditioned once with water under gravity flow, followed by conditioning with methanol. Sample tube was then connected to flushed cartridge. Vacuum pressure and flow rate were adjusted to 10 mmHg and 5–7 ml min^–1^, respectively. Waste solvent was collected in waste container. Cartridge was dried under maximum pressure and residues were eluted with methanol.

### Allelochemical Profiling

#### Preliminary Identification of Allelochemicals With High-Performance Liquid Chromatography

Methanolic fractions of crude extracts (castor, artemisia, wheat, and sorghum) were used for identification of phenolic acids and flavonoids as described by Hussain et al. (2013). Chemicals for standards and mobile phase were purchased from Sigma-Aldrich (United States). Phenolic compound analyses were performed with system LC-10A, including C18 column (i.d. 250 × 4.6 mm, pore size 5 μm, Phenomenex, Jupiter 5u C18, 300A). UV-Vis detection system was used with class LC-10 software (Shimadzu RF-530, fluorescence monitor). Column was thermostatically controlled at 30°C temperature. Standards for flavonoids (rutin, quercetin, myricetin, kaempferol, and catechin) were prepared with a concentration of 10 μg ml^–1^ (acetonitrile). Phenolic acids standards (syringic acid, *p*-hydroxy benzoic acid, vanillic acid, ferulic acid, gallic acid, chlorogenic acid, *p*-coumaric acid, caffeic acid, and sinapic acid) were prepared at concentration of 20 μg ml^–1^ (methanol). Mobile phase used for identification and quantification of flavonoids was composed of acetonitrile, methanol (80:20), and acetic acid (3%). For analysis of phenolic acids, mobile phase consisted of mixture of acetonitrile, distilled water (10:88), and acetic acid (2%), followed by its filtration through filtration assembly (0.45 μm × 47 mm). Sonication of mobile phase was done for 10 min (Model-EYELA, Toyota). Column was purged to remove initial mobile phase from the system before application of standards. Flow rate and pressure (maximum) of solvent were set as 1 ml min^–1^ and 250 kgf cm^–2^, respectively. Each standard was injected into the micro injector with a volume of 20 μl. Retention times of standards were noted for reference. Peak detected through UV detector was obtained, and peak area was identified for quantification of phenolic compounds in pure methanolic fractions. Samples were analyzed in three replicates.

#### Liquid Chromatography–Mass Spectrometric Analysis for Allelochemical Identification

The detailed profiling of allelochemicals present in each allelopathic extract was performed according to protocols as described by Kang et al. (2016), with minor modifications. Liquid chromatography–mass spectrometry (LC-MS) analysis was executed in Agilent triple quadrupole system. Compounds were separated on column compartment (model: G1776A, dimensions: 160 × 435 × 436 mm, temperature: 40°C). Volume of injection was 5.00 μl. Mobile phase contained solvent A (100% acetonitrile) and solvent B (100% water). Flow rate of mobile phase was 0.3 ml min^–1^. HPLC gradient included 0–4 min, 2% A, 98% B; 4–7 min, 20% A, 80% B; 7–14 min, 90% A, 10% B; 15 min, 90% A, 10% B; and 15.10 min, 2% A and 98% B. MS was conducted in electrospray ionization (ESI)-positive mode by nitrogen gas under the following conditions: gas temperature 350°C, gas flow 11 L min^–1^, nebulizer 50 psi, and capillary voltage 4,500 V. Identification of allelochemical was determined by using the National Institute of Standards and Technology’s (NIST17) database.

### Statistical Analysis

The experiments for seed germination, seedling growth, and phytochemical analysis were conducted in completely randomized design (CRD) with three replicates. Each experiment was repeated thrice. Treatment values were normalized to methanol prior to analysis. Results were analyzed with two-way multivariate ANOVA using general linear model. Variables were transformed where and when required for data normalization. Percentage data were arcsine transformed, while the rest of the variables were log transformed for normality. Transformed variables were back transformed, and data were presented as mean** ±** standard errors of means (SEM). Significance of obtained results was analyzed with Tukey’s honestly significant difference (HSD) test at probability level *P* < 0.05 with SPSS v. 23 (IBM, United States).

## Results

### Inhibitory Effects of Methanolic Extracts on Weed Seed Germination

**FIGURE 1:**
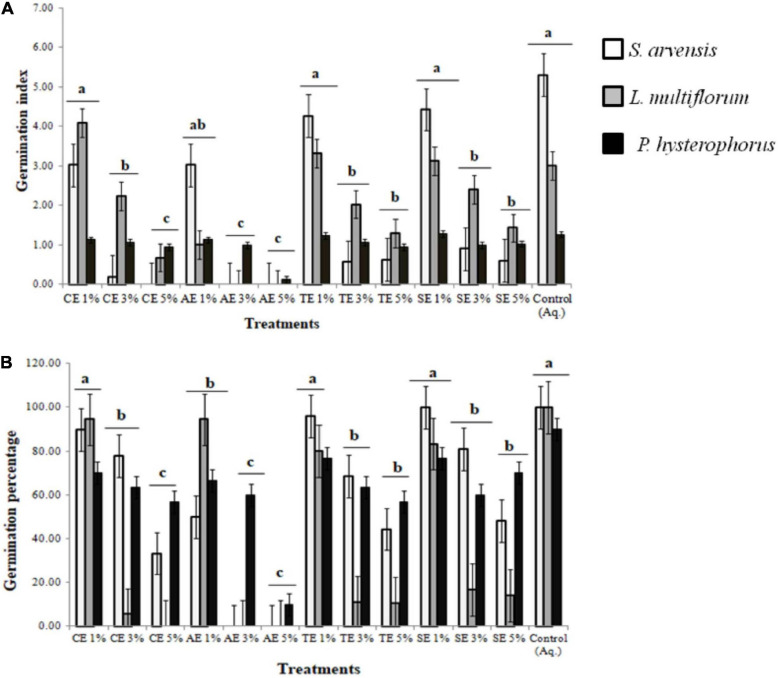
Effect of methanolic extracts of castor (CE), artemisia (AE), wheat (TE), and sorghum (SE) on **(A)** germination index (GI) and **(B)** germination percentage (G%) of *Sinapis arvensis*, *Lolium multiflorum*, and *Parthenium hysterophorus.* Data are presented as mean ± SEM (*n* = 30). Bar values with different letters differ significantly at *P* < 0.05. One percent of all extracts did not show any significant inhibition against weeds, whereas 3 and 5% AE along with 5% of all the other extracts significantly reduced GI and G%, whereby inducing 40–100% germination inhibition in all weeds.

The potential allelopathic contribution of castor (*Ricinus communis*), artemisia (*Artemisia santolinifolia*), wheat (*Triticum aestivum* L.), and sorghum (*Sorghum bicolor*) extracts revealed that 3 and 5% concentration significantly reduced seed germination index and germination percentage. The 5% castor extract induced 100, 70, and 40% germination inhibition in *Sinapis arvensis*, *Lolium multiflorum*, and *Parthenium hysterophorus*, respectively (Supplementary Figures 1–3). Artemisia extracts (3 and 5%) were able to completely inhibit the germination of *S. arvensis* and *L. multiflorum*, while 90% inhibition of *P. hysterophorus* was observed. Wheat and sorghum extracts were able to induce 60–90% and 30–80% germination inhibition in all weeds, respectively. Solvent and aqueous controls had no negative effect on germination index or percentage and seeds sprouted in a normal time period (Figures 1A,B).

### *In vitro* Evaluation of Seedling Growth of Weeds

**FIGURE 2:**
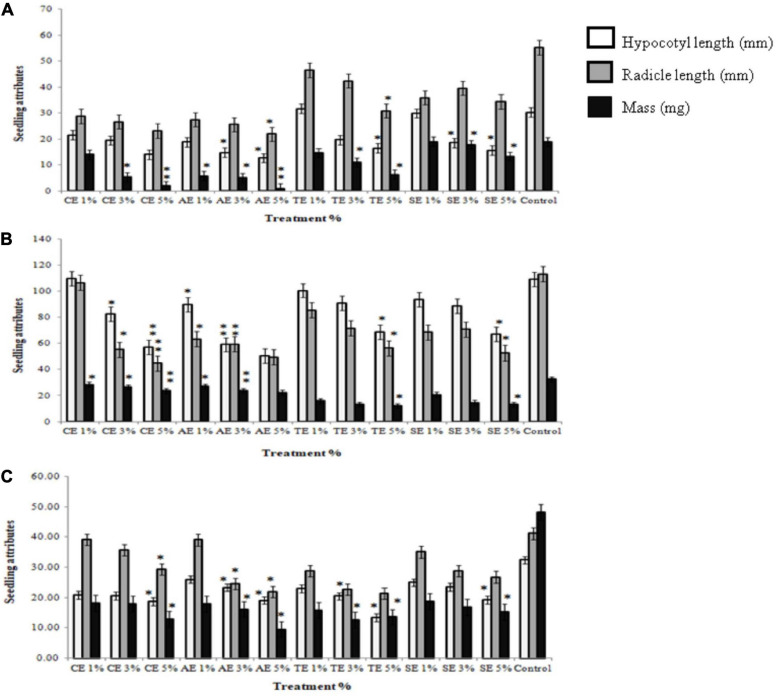
Seedling attributes of weeds after treatment with castor extract (CE), artemisia extract (AE), wheat extract (TE), sorghum extract (SE), and aqueous control. **(A)** Effect on *Sinapis arvensis*; **(B)** effect on *Lolium multiflorum*; **(C)** effect on *Parthenium hysterophorus*. Values are presented as mean ± SEM (*n* = 30). Bar values with asterisks differ significantly from control at *P* < 0.05.

To determine the seedling growth rate, radicle length, hypocotyl length, and biomass of weed seedlings were measured. Results illustrated that 3 and 5% extracts significantly reduced average root and shoot length of all weeds as well as the relative seedling growth. Sorghum extract showed the least significant effect on all weeds. No reduction in seedling growth was observed in control treatments (Supplementary Figures 4–8). Apart from stunting growth attributes, extract treatments also exhibited a bleaching effect in weeds. Any effect of similar concentrations of methanol was normalized to aqueous control (Figures 2A–C).

### Phytochemical Analysis of Allelopathic Extracts

#### Total Phenolic Acid Content

TPC of 1, 3, and 5% castor, artemisia, wheat, and sorghum extracts were quantified. The regression equation for calibration curve of gallic acid (6.25–100 μg ml^–1^) was y = 0.0045x + 0.0623 with R^2^ = 0.9656 (Supplementary Figure 9A).

Five percent castor extract had the highest phenolic acid content (349.09 ± 1.35 mg GAE/100 g) followed by artemisia (112.93 ± 1.65 mg GAE/100 g), wheat (101.47 ± 2.09 mg GAE/100 g), and sorghum (85.85 ± 1.19 mg GAE/100 g). TPC of different concentrations of each extract is mentioned in Table 1.

**TABLE 1:** Total phenolic content in various concentrations of castor, artemisia, wheat, and sorghum extracts (CE, AE, TE, and SE, respectively).

Extract concentration	Phenolic content (mg of gallic acid equivalent/100 g dry material)	Flavonoid content (mg of quercetin equivalent/100 g dry material)
CE 1%	77.79 ± 3.06	129.0 ± 1.89
CE 3%	256.75 ± 3.89	326.50 ± 1.05
CE 5%	349.09 ± 1.35	697.0 ± 2.40
AE 1%	39.28 ± 0.74	101.83 ± 2.12
AE 3%	101.95 ± 2.01	405.67 ± 1.09
AE 5%	112.93 ± 1.65	896.67 ± 2.36
TE 1%	49.74 ± 2.89	395.83 ± 7.10
TE 3%	54.06 ± 1.44	502.00 ± 2.84
TE 5%	101.47 ± 2.09	583.33 ± 1.73
SE 1%	59.73 ± 1.44	361.17 ± 1.76
SE 3%	58.79 ± 3.02	396.33 ± 5.07
SE 5%	85.85 ± 1.19	571.17 ± 3.19

*Values are presented as mean ± SEM (n = 3).*

#### Total Flavonoid Content

TFC of 1, 3, and 5% castor, artemisia, wheat, and sorghum extracts was quantified. The regression equation for calibration curve of quercetin (100–1,000 μg ml^–1^) was y = 0.0002x + 0.0884 with R^2^ = 0.9689 (Supplementary Figure 9B).

Content of total flavonoids was observed to be the highest in 5% artemisia extract (896.67 ± 2.36 mg QE/100 g) followed by castor (697.0 ± 2.40 mg QE/100 g), wheat (583.33 ± 1.73 mg QE/100 g), and sorghum (571.17 ± 3.19 mg QE/100 g). The range of TFC in all dilutions is given in Table 1.

### *In vivo* Effect on Morphology and Chlorophyll Content of Weeds

In soil pot experiment, all the test species of weeds permanently wilted and showed a faded effect after the treatment’s applications. *S. arvensis* seedlings became flaccid after 2 h of extract application; however, *L. multiflorum* and *P. hysterophorus* showed reduction in seedling growth after 24 and 72 h of fraction application, respectively. Permanent growth inhibition was observed after 5 days of experiment with three applications of extracts on alternate days. The most significant effect was demonstrated with artemisia extract against all weeds. *P. hysterophorus* showed the least phenotypic changes in comparison with control seedlings.

**FIGURE 3:**
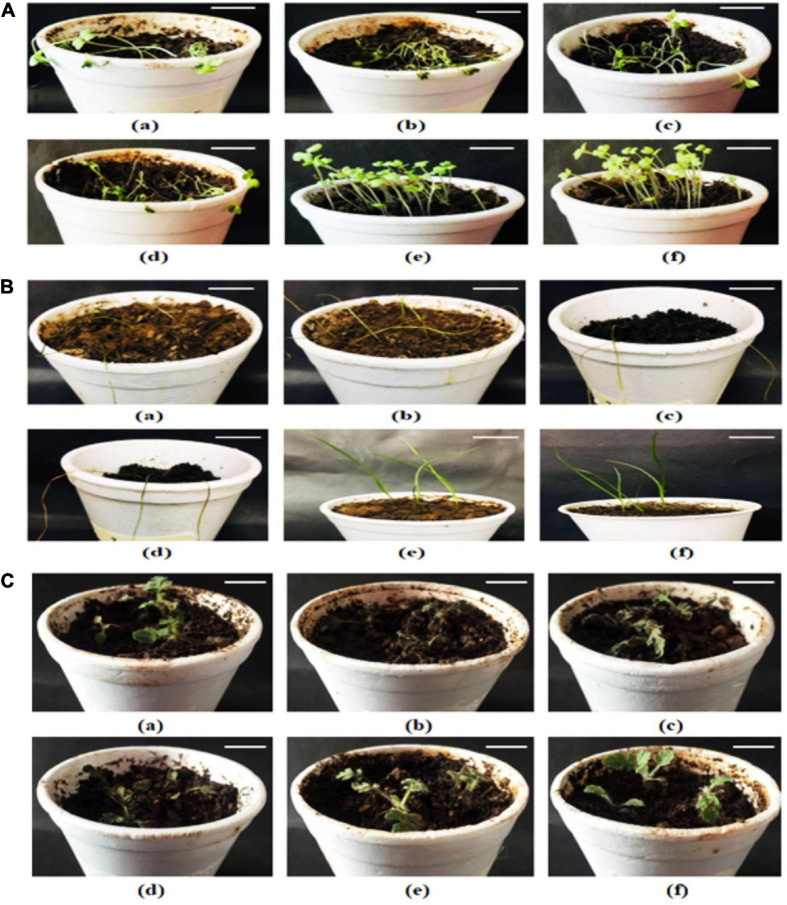
Effect of 20% extracts on weed growth **(A)** *Sinapis arvensis*, **(B)** *Lolium multiflorum*, and **(C)** *Parthenium hysterophorus*. In each panel, (a–f) indicate effect of extracts of castor, artemisia, sorghum, wheat, 20% solvent control, and aqueous control, respectively, on each weed. *S. arvensis* showed the most significant growth suppression against all extracts and permanently wilted after 2 h of treatment application. *P. hysterophorus* demonstrated the least sensitivity against treatments (bar = 7.5 cm).

**FIGURE 4:**
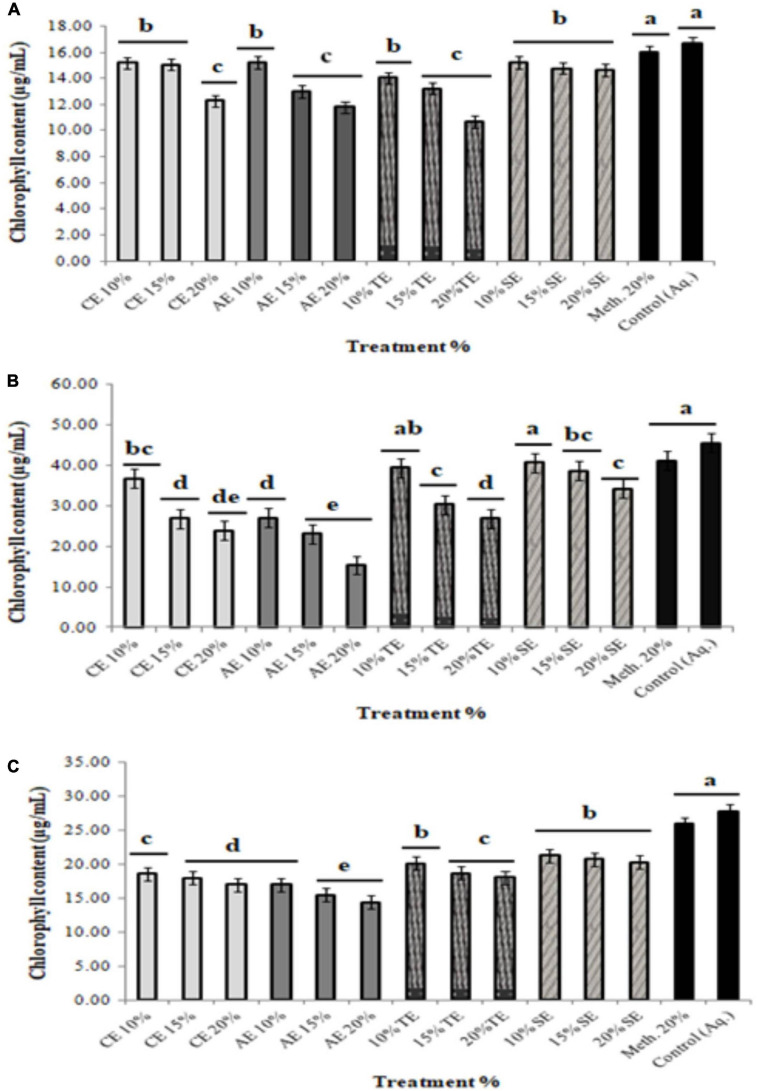
Total chlorophyll content of weeds treated with 10, 15, and 20% extracts of castor (CE), artemisia (AE), wheat (TE), sorghum (SE), methanolic control (20%), and aqueous control. **(A)** *Sinapis arvensis*, **(B)** *Lolium multiflorum*, and **(C)** *Parthenium hysterophorus*. Values are presented as mean ± SEM (*n* = 3). Bar values with different letters differ significantly at *P* < 0.05.

Since chlorophyll content determines the photosynthetic ability and the growth pattern of the plants; thus, 10 and 15% extract application did not affect the chlorophyll content significantly, whereas 20% of all allelopathic extracts drastically reduced chlorophyll content of the weeds, resulting in dead seedlings (Figure 3). Seedlings treated with methanolic control (20%) and aqueous control demonstrated normal growth pattern (Figures 4A–C).

### Biochemical Analysis of Weeds

**FIGURE 5:**
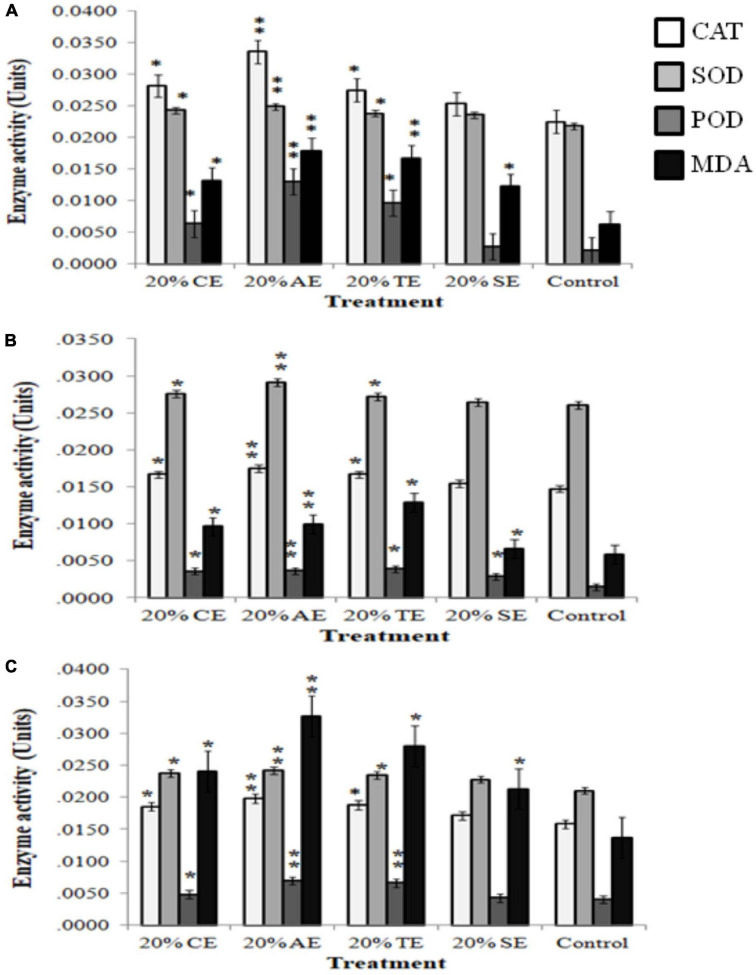
Activity of antioxidant enzymes [catalase (CAT), superoxide dismutase (SOD), and peroxidase (POD)] and lipid peroxidation [malondialdehyde (MDA)] in response to 20% castor (CE), artemisia (AE), wheat (TE), sorghum (SE), and aqueous control in **(A)** *Sinapis arvensis*, **(B)** *Lolium multiflorum*, and **(C)** *Parthenium hysterophorus*. Data are presented as mean ± SEM (*n* = 3). Bar values with asterisks differ significantly from control at *P* < 0.05. Twenty percent of CE, AE, and TE showed significant increase in enzyme activity as well as lipid peroxidation as compared with that of control.

To evaluate whether the negative effects of 20% extracts were caused by excessive production of reactive oxygen species (ROS) and altered membrane structure, activity of few antioxidant enzymes (CAT, SOD, and POD) that regulate ROS levels along with membrane damage by lipid peroxidation was investigated. Results indicated that activity of these enzymes significantly increased in weeds, where 20% AE depicted the most significant increase (20–80%) in CAT, SOD, POD, and lipid peroxidation, in all tested weeds. CE and TE also altered enzymatic and MDA levels, whereas SE had no significant effect on these enzymes while increasing MDA level in all weeds (Figures 5A–C).

### Preliminary Identification of Allelochemicals With High-Performance Liquid Chromatography

Methanolic fraction of extracts (from SPE) revealed the presence of various phenolic compounds and flavonoids against the standards employed. Flavonoids identified in extracts included rutin, quercetin, myricetin, kaempferol, and catechin. Concentration of flavonoids ranged from 2.31 to 3,869.36 μg ml^–1^, with rutin (3,869.36 μg ml^–1^) and quercetin (1,114.54 μg ml^–1^) having the highest concentration in wheat and artemisia, respectively.

Identified phenolic acids included gallic acid, vanillic acid, *p*-hydroxy benzoic acid, chlorogenic acid, *p*-coumaric acid, caffeic acid, and sinapic acid, with a range of 0.02–856.35 μg ml^–1^ among all extracts (Table 2). In phenolic acids, gallic acid had the highest amount in all extracts. Kaempferol, catechin, and syringic acid were not found in artemisia extract, while *p*-hydroxy benzoic acid was absent in sorghum extract.

**TABLE 2:** Phenolic acids and flavonoids, determined in methanolic extracts of castor (CE), artemisia (AE), wheat (TE), and sorghum (SE) by HPLC.

**Compounds**	**Concentration (μg/ml)**			
	**CE**	**AE**	**TE**	**SE**
Rutin	2120.6 ± 3.78	5.96 ± 0.42	3869.36 ± 67.88	483.83 ± 3.78
Quercetin	2.29 ± 0.26	1114.54 ± 40.10	2.19 ± 0.30	0.15 ± 0.00
Myricetin	58.2 ± 1.89	129.711 ± 43.38	30.82 ± 1.47	43.55 ± 0.32
Kaempferol	7.56 ± 0.72	ND	4.02 ± 0.57	14.98 ± 0.57
Catechin	62.99 ± 1.83	ND	2.31 ± 0.33	17.2 ± 0.61
Gallic acid	856.35 ± 39.75	462.77 ± 19.08	37.98 ± 0.25	283.37 ± 0.69
Vanillic acid	0.76 ± 0.15	31.85 ± 0.59	0.015 ± 0.00	0.19 ± 0.00
Syringic acid	4.29 ± 0.45	ND	0.06 ± 0.00	0.11 ± 0.00
Ferulic acid	0.17 ± 0.02	0.98 ± 0.06	0.07 ± 0.01	0.2 ± 0.05
*p*-hydroxy benzoic acid	25.01 ± 1.72	1.45 ± 0.15	1.61 ± 0.09	ND
Chlorogenic acid	56.36 ± 2.17	33.62 ± 1.09	12.99 ± 0.49	4.25 ± 0.63
*p*-coumaric acid	34.71 ± 0.65	29.38 ± 1.15	16.21 ± 0.91	3.79 ± 0.47
Caffeic acid	0.02 ± 0.00	18.53 ± 0.87	0.04 ± 0.00	0.55 ± 0.02
Sinapic acid	7.42 ± 1.22	7.25 ± 0.69	0.06 ± 0.01	0.05 ± 0.02

*ND, not determined; HPLC, high-performance liquid chromatography. Values are presented as mean ± SEM (n = 3).*

### Liquid Chromatography–Mass Spectrometric Analysis for Allelochemical Identification

**FIGURE 6:**
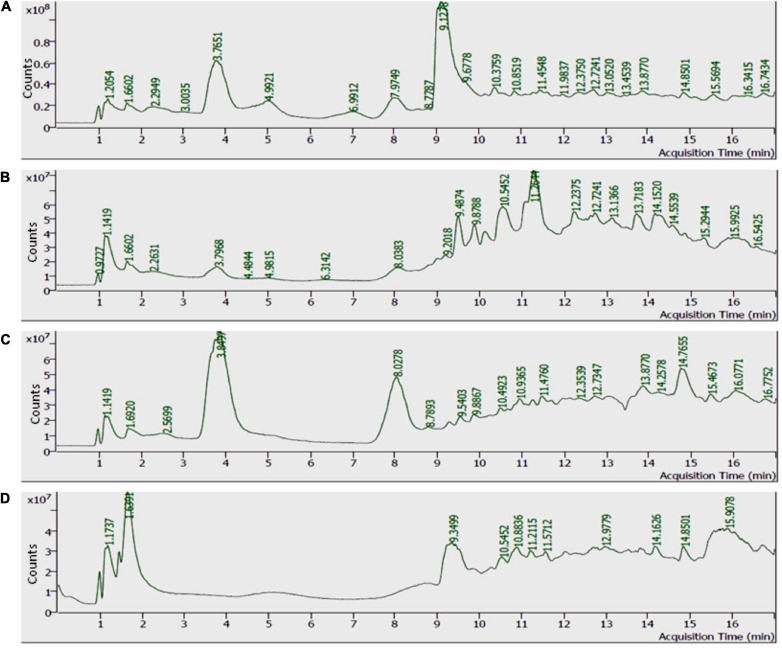
Total ion chromatogram (TIC) of methanolic extracts. **(A)** Castor extract, **(B)** artemisia extract, **(C)** wheat extract, and **(D)** sorghum extract.

Scanning and characterization of allelochemicals and their derivates in individual allelopathic fraction were performed by LC-MS (Figure 6).

Analyses illustrated the presence of various aromatic compounds and their derivates, which were explicitly unexplored so far. Among 120, 113, 90, and 50 compounds annotated in CE, AE, TE, and SE, respectively, the majority belonged to alkaloids, phenolic acids, and flavonoids derivates (Supplementary Figure 10 and Supplementary Tables 1–4). Quantity and classes of compounds in each fraction correlate with their bioactivity. Abundantly present metabolites as depicted by percentage peak area included 4-amino-3-methoxypyrazolo[3,4-*d*]pyrimidine; propanamide, 2-amino-3-phenyl and fumaric acid, monoamide, *N*-methyl-*N*- phenyl-, 4-chloro-2-methylphenyl ester in CE; 2,4,6-trimethoxybenzonitrile; dimethylmalonic acid, 3-phenylpropyl tridecyl ester, and cyclopropanecarboxamide, *N*-[4-(3-methyl-1*H*-pyrazol-1-yl)phenyl] in AE; 5,6,7,8-tetrahydroindolizine; fumaric acid, monoamide, *N*-methyl-*N*- phenyl-, 4-chloro-2-methylphenyl ester, and propanamide, 2-amino-3-phenyl in TE and propanamide, 2-amino-3-phenyl; 1-methyl-3-phenylpiperazine and quinoxaline, 5-methyl in SE (Tables 3–6). These major compounds belong to alkaloid, phenolic acid, and flavonoid groups and are reported as novel derivates in these plant species.

**TABLE 3:** Most abundant compounds identified by LC-MS in castor extract.

Compound identified	Molecular formula	RT (min)	Area %	Match score	Compound class
Purine-2,6-dione, 1,3-dimethyl-7-(2-oxo-2-phenylethyl)-8-(piperidin-1-yl)-3,7-dihydro-	C_2__0_H_2__3_N_5_O	1.2054	3.57	50.0	Alkaloid
Decahydronaphtho[2,3-*b*]furan-2-one, 3-pyrrol[2-(4-fluorophenyl)ethylamino]methylmorpho-8a-methyl-5-methylene-	C_2__3_H_3__0_FNO_2_	1.6602	2.53	48.2	Alkaloid
Propanamide, 2-amino-3-phenyl	C_9_H_1__2_N_2_O	3.7651	23.74	74.6	Phenolic acid
6-Hydroxy-3′-methoxyflavone	C_1__6_H_1__2_O_4_	4.7382	1.85	55.0	Flavonoid
2(1*H*)-Pyridinone, 1-cyclohexyl-3,4,5,6-tetramethyl-	C_1__5_H_2__3_NO	6.9912	2.59	51.9	Alkaloid
6,7-Epoxypregn-4-ene-9,11,18-triol-3,20-dione, 11,18-diacetate	C_2__5_H_3__2_O_8_	7.0546	1.62	50.1	Flavonoid
Fumaric acid, monoamide, *N*-methyl-*N*- phenyl-, 4-chloro-2-methylphenyl ester	C_1__8_H_1__6_ClNO_3_	7.9749	6.90	62.9	Phenolic acid
4-Amino-3-methoxypyrazolo[3,4-*d*]pyrimidine	C_6_H_7_N_5_O	9.1278	41.69	56.9	Alkaloid
Benzoic acid, 4- propyl-, octadecyl ester	C_2__8_H_4__8_O_2_	9.6778	2.52	38.2	Phenolic acid
2,6-Difluoro-3-methylbenzoic acid, nonadecyl ester	C_2__7_H_4__4_F_2_O_2_	11.4548	0.73	34.2	Phenolic acid
3*H*-Naphtho[2,3-*b*]furan-2-one, 3-[[2-(4-fluorophenyl)ethylamino]methyl]-5,8a-dimethyl-3a,5,6,7,8,8a,9,9a-octahydro-	C_2__3_H_3__0_FNO_2_	12.3750	0.66	35.0	Flavonoid
Pyridine-3-carbonitrile, 1,4-dihydro-2-amino-1-(3-cyano-4-ethyl-5-methyl-2-thienyl)-4,4-bis(trifluoromethyl)-2-methyl-	C_1__7_H_1__4_F_6_N_4_S	12.7241	1.28	38.3	Alkaloid
4-Hydroxymandelic acid, ethyl ester, di-PFP	C_1__6_H_1__0_F_1__0_O_6_	14.8501	0.99	39.6	Phenolic acid
6*H*-Pyrazolo[3,4-*H*]quinazoline, 2-amino-9-methyl-7-(pyridin-2-yl)-5,7-dihydro-	C_1__5_H_1__4_N_6_	15.5694	1.28	32.6	Alkaloid

*RT, retention time; LC-MS, liquid chromatography–mass spectrometry.*

**TABLE 4:** Most abundant compounds identified by LC-MS in artemisia extract.

Compound identified	Molecular formula	RT (min)	Area %	Match score	Compound class
Benzeneacetic acid, 2-phenylethyl ester	C_1__6_H_1__6_O_2_	1.1419	3.95	49.5	Phenolic acid
Dimethylmalonic acid, 3-phenylpropyl tridecyl ester	C_2__7_H_4__4_O_4_	1.1948	9.74	34.1	Phenolic acid
3,11-Diazatricyclo[7.3.1.0(3.8)]trideca-5,7-dien-4-one, 11-(4-hydroxy-2-butynyl)-	C_1__5_H_1__8_N_2_O_2_	1.6602	4.46	40.1	Alkaloid
Benzo[*a*]phenanthridin-4(3*H*)-one, 1,2,5,6-tetrahydro-2,2-dimethyl-5-(3-hydroxyphenyl)-	C_2__5_H_2__3_NO_2_	2.2631	2.38	49.0	Alkaloid
Propanamide, 2-amino-3-phenyl	C_9_H_1__2_N_2_O	3.7968	5.00	59.5	Phenolic acid
Acetamide, *N*-[1,2,3,4-tetrahydro-1-(2-furoyl)-2-methyl-4-quinolinyl]-N-phenyl-	C_2__3_H_2__2_N_2_O_3_	8.0383	2.91	49.0	Alkaloid
2-Formylfuro[2,3-b]pyridin-3-yl acetate	C_1__0_H_7_NO_4_	9.4874	5.58	41.0	Alkaloid
Butane, 1,4-bis(9,10-dihydro-9-methylanthracen-10-yl)-	C_3__4_H_34_	9.8788	2.28	40.4	Phenolic acid
Cyclopropanecarboxamide, *N*-[4-(3-methyl-1*H*-pyrazol-1-yl)phenyl]-	C_1__4_H_1__5_N_3_O	10.5452	8.16	58.6	Alkaloid
Cyclopropanecarboxylic acid, 3-(2,2-dichloroethenyl)-2,2- dimethyl-, (3-phenoxyphenyl)methyl ester, *cis*-	C_2__1_H_2__0_Cl_2_O_3_	11.1057	1.31	30.9	Phenolic acid
2,4,6-Trimethoxybenzonitrile	C_1__0_H_1__1_NO_3_	11.2644	22.78	51.6	Phenolic acid
Isoquinoline, 1,2,3,4-tetrahydro-1-(3-fluorophenyl)-6,7-dimethoxy-	C_1__7_H_1__8_FNO_2_	12.2375	5.68	38.8	Alkaloid
Benzoic acid, 3,4- dimethoxy-, 4-[ethyl[2-(4-methoxyphenyl)-1-methylethyl]amino]butyl ester	C_2__5_H_3__5_NO_5_	12.7241	3.15	40.3	Phenolic acid
4-(4-Chloro-phenyl)-1-(tetrahydro-furan-2-ylmethyl) 1,4-dihydro-pyridine-3,5-dicarboxylic acid dimethyl ester	C_2__0_H_2__2_ClNO_5_	13.1366	2.91	38.8	Alkaloid
Lanostan-12-one	C_3__0_H_5__2_O	13.7183	3.56	49.0	Flavonoid
L-Homophenylalanine, *N*,*N*-bis(3-methylbutyl)-, 3-methylbutyl ester	C_2__5_H_4__3_NO_2_	15.8761	2.28	35.9	Phenolic acid
Thiophene-3-carbonitrile, 4-amino-5-(4-fluorobenzoyl)-2-methylamino-	C_1__3_H_1__0_FN_3_OS	15.9925	2.92	33.0	Alkaloid

*RT, retention time; LC-MS, liquid chromatography–mass spectrometry.*

**TABLE 5:** Most abundant compounds identified by LC-MS in wheat extract.

Compound identified	Molecular formula	RT (min)	Area %	Match score	Compound class
Thiosulfuric acid *S*-2-[[2-[[4-methyl-2-quinolyl]oxy]ethyl]amino]ethyl ester	C_1__4_H_1__8_N_2O_4S_2_	0.9727	1.04	46.3	Alkaloid
Acetic acid, 2-phenylethyl ester	C_1__0_H_1__2_O_2_	1.1419	1.29	38.8	Phenolic acid
(*S*)-7-Bromo-3-isobutyl-2-(2-methylbenzyl)-3,4-dihydro-2*H*-benzo[*b*][1,4,5]oxathiazepine 1,1-dioxide	C_2__0_H_2__4_BrNO_3_S	1.2054	4.39	33.1	Alkaloid
Prednisolone	C_2__1_H_2__8_O_5_	2.5699	2.22	47.9	Flavonoid
Propanamide, 2-amino-3-phenyl	C_1__9_H_1__2_N_2_O	3.7440	22.34	70.3	Phenolic acid
5,6,7,8-Tetrahydroindolizine	C_8_H_1__1_N	3.8497	29.59	52.4	Alkaloid
Fumaric acid, monoamide, *N*-methyl-*N*- phenyl-, 4-chloro-2-methylphenyl ester	C_1__8_H_1__6_ClNO_3_	8.0278	22.91	69.0	Phenolic acid
2-Hydroxyethylflurazepam, tert-butyldimethylsilylether	C_2__3_H_2__8_ClFN_2_O_2_Si	9.8867	0.62	42.0	Alkaloid
2-Propenamide, 2-cyano-*N*,*N*-dimethyl-3-[4-[[4-(dimethylamino)phenyl]azo]phenyl]-	C_2__0_H_2__1_N_5_O	10.9365	1.83	40.6	Phenolic acid
Benzoic alcohol, 2-hydroxy-3,5-dinitro-	C_7_H_6_N_2_O_6_	11.2538	0.72	32.3	Phenolic acid
2,6-Difluoro-3-methylbenzoic acid, heptadecyl ester	C_2__5_H_4__0_F_2_O_2_	11.4760	0.97	34.1	Phenolic acid
*N*-(2-Fluoro-phenyl)-2-methoxy-4-methylsulfanylbenzamide	C_1__5_H_1__4_FNO_2_S	13.8770	1.41	34.3	Phenolic acid
4-Hexen-3-one oxime, o-[(pentafluorophenyl)methyl]-	C_1__3_H_1__2_F_5_NO	15.4673	0.84	35.1	Phenolic acid
1(3*H*)-Isobenzofuranone, 3,3′-(4-methoxy-1,3-phenylene)bis[3-(4-methoxyphenyl)-	C_3__7_H_2__8_O_7_	16.0771	3.04	29.3	Flavonoid

*RT, retention time; LC-MS, liquid chromatography–mass spectrometry.*

**TABLE 6:** Most abundant compounds identified by LC-MS in sorghum extract.

Compound identified	Molecular formula	RT (min)	Area %	Match score	Compound class
Thiosulfuric acid S-2-[[2-[[4methyl-2-quinolyl]oxy]ethyl]amino]ethyl ester	C_1__4_H_1__8_N_2_O_4_S_2_	0.9883	3.03	44.5	Alkaloid
1-Methyl-3-phenylpiperazine	C_1__1_H_1__6_N_2_	1.1737	17.20	39.9	Alkaloid
Quinine	C_2__0_H_2__4_N_2_O_2_	1.4487	5.98	43.5	Alkaloid
Propanamide, 2-amino-3-phenyl	C_9_H_1__2_N_2_O	1.6391	45.91	66.7	Phenolic acid
Quinoxaline, 5-methyl-	C_9_H_8_N_2_	9.3499	8.85	54.0	Alkaloid
Isatin biscresol, 3TMS derivative	C_3__1_H_4__3_NO_3_Si_3_	10.5452	1.55	38.0	Alkaloid
4-Azatricyclo[5.2.1.0(2,6)]decane-3,5-dione, 4-[4-(2-methylphenoxy)phenyl]-	C_2__2_H_2__1_NO_3_	10.8836	5.25	45.3	Alkaloid
Benzene, 1-[2-bromo-1-(methoxydiphenylmethyl)ethyl]-4-methoxy-	C_2__3_H_2__3_BrO_2_	11.5712	1.75	31.0	Phenolic acid
Pyridine-3-carbonitrile, 1,4-dihydro-2-amino-1-(3-cyano-4-ethyl-5-methyl-2-thienyl)-4,4-bis(trifluoromethyl)-2-methyl-	C_1__7_H_1__4_F_6_N_4_S	12.7029	1.36	35.3	Alkaloid
Flumequine, *tert*-butyldimethylsilyl ester	C_2__0_H_2__6_FNO_3_Si	12.9779	1.88	31.5	Alkaloid
4-Hydroxymandelic acid, ethyl ester, di-PFP	C_1__6_H_1__0_F_1__0_O_6_	14.8501	2.25	37.2	Phenolic acid
2,4-Dimethoxy-4b,5,6,7,8,8a,9,10-octahydrophenanthren-1-ol-5,10-dione-7-acetic acid, methyl ester	C_1__9_H_2__2_O_7_	15.9078	4.99	32.5	Alkaloid

*RT, retention time; LC-MS, liquid chromatography–mass spectrometry.*

## Discussion

**FIGURE 7:**
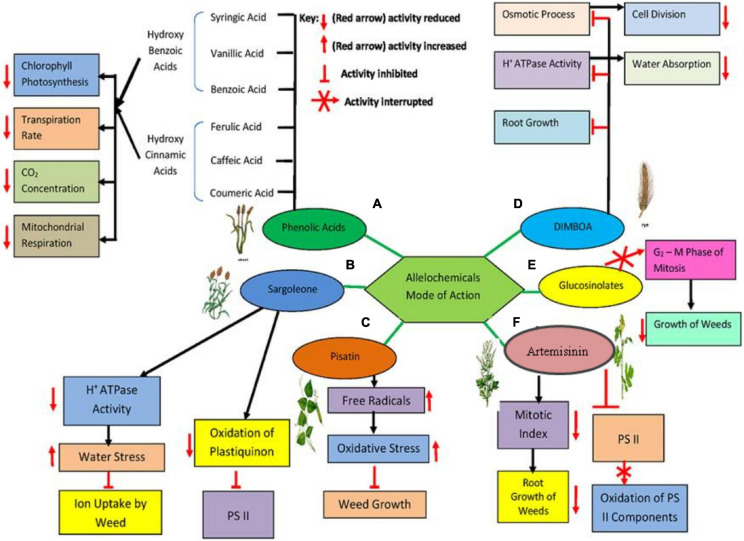
A schematic diagram showing the mode of actions of selected allelochemicals to interfere with various biochemical pathways of weeds. **(A)** Interference of phenolics, i.e., hydroxyl benzoic and hydroxyl cinnamic acids, to suppress entire photosynthetic activity as well as mitochondrial respiration. **(B)** Reduction in ion uptake of weeds due to restrained ATPase activity and increased water stress along with blockage of photosystem II (PSII) induced by sorgoleone, an important allelochemical in sorghum. **(C)** Suppressed weed growth as a result of increased oxidative stress in weeds stimulated by pisatin occurring in pea. **(D)** Blockage of ATPase activity, osmotic process, and resulting repression of weed’s root growth due to DIMBOA from wheat and maize. **(E)** Glucosinate (allelochemical in mustard plant) induced reduction of mitotic cell cycle (G_2_-M phase). **(F)** Diminished mitotic index and activity of PSII intervened by artemisinin, the most significant allelochemical in artemisia. All these allelochemicals released from potent plants present them as a powerful source of natural herbicides.

Allelopathy has a pertinent significance for sustainable, ecological, and integrated weed management systems (Jabran et al., 2015). The advantages of allelochemicals lie in their versatility of not only being inhibitor of weed growth but also being eco-sustainable. The secondary phytotoxic metabolites released by plants have been focused in agriculture system as a multidisciplinary science for sustainable production (Amb and Ahluwalia, 2016). There are no generic systemic of allelochemicals in crops system, and henceforth, phytotoxic potential varies from plant-to-plant species. These differences across species and allelopathic activities of different phytochemicals have uncovered many aspects of future investigation. Majority of the allelochemicals are completely or partly soluble in water, which makes their application easier without addition of surfactants (Dayan et al., 2009). The structural chemistry of such natural compounds makes them environment friendly as compared with artificially synthesized. Moreover, these allelochemicals are composed of higher content of oxygen and nitrogen, with a less significant amount of heavy and unnatural rings (Soltys et al., 2013). Such attributes reduce their half-life, preventing their accumulation in the environment and ultimately decreasing their toxicity to non-targeted organisms while increasing their efficacy against weeds. The mechanism of suppression of weed growth as affected by few of such allelochemicals’ activity has been summarized (see Figure 7), wherein the most significant compounds including phenolic acids, artemisinin, DIMBOA (2,4-dihydroxy-7-methoxy-1,4-benzoxazin-3-one), and sorgoleone are involved in suppressing photosynthetic activity, elevating water stress, and declining cell division to reduce the overall weed growth and development.

In this study, the phytotoxic potential of methanolic extracts of castor (*R. communis*), artemisia (*A. santolinifolia*), wheat (*T. aestivum*), and sorghum (*S. bicolor*) was used in bioassay-based detection of allelopathins to control the invasion of weeds of wheat in an eco-friendly manner.

Germination index and growth suppression are preliminary determinants of the allelopathic activity of extracts (Puig et al., 2018). Initially, the *in vitro* phytotoxicity was assessed by the inhibition of weeds’ (*S. arvensis*, *L. multiflorum*, and *P. hysterophorus*) germination rate and growth suppression in response to application of methanolic extracts of castor, artemisia, wheat, and sorghum, which was found to be dose dependent (3–5% concentration). Phytotoxicity of artemisia extracts was more pronounced leading up to 100% germination inhibition in *S. arvensis* and *L. multiflorum* while 90% in *P. hysterophorus*. The castor extract produced 100, 70, and 40% germination inhibition in *S. arvensis*, *L. multiflorum*, and *P. hysterophorus*, respectively. Germination reduction may be associated with allelochemical interference due to inhibition of amylases and gibberellins, hence modifying the process of mobilization of reserves for embryo development (Singh et al., 2009). Hypocotyl and radicle growth was reduced significantly in response to 3 and 5% extract application in comparison with solvent and aqueous controls. Aslani et al. (2014) reported that seed emergence and early growth of seven test plants, including tomato, lettuce, cucumber, rice, and its weeds, were inhibited with increasing concentrations of methanolic extract of *Tinospora tuberculata*. Similar results were demonstrated by Han et al. (2008) and Sodaeizadeh et al. (2009) where phytotoxic effects of ginger (*Zingiber officinale*) and harmel (*Peganum harmala*) aqueous extracts were concentration dependent against early growth of *Avena fetua* and *Convolvulus arvensis*.

Environmental conditions such as biotic or abiotic factors contribute toward structural modification of allelochemicals, when applied in soil (Jilani et al., 2008); thus, the initial concentrations of extracts play a predominant role in determining its phytotoxicity. For this purpose, we used 10, 15, and 20% plant extracts to evaluate their toxicity level for growth suppression in soil experiments. The 20% concentration of all the extracts demonstrated permanent wilting in *S. arvensis*, *L. multiflorum*, and *P. hysterophorus* after 2, 24, and 72 h, respectively. In response to treatment application, a bleaching effect in leaves of *S. arvensis* and *L. multiflorum* was observed. Therefore, total chlorophyll content of all weed seedlings was decreased significantly with increased extract concentration. The reduction of chlorophyll levels in lettuce was found to be dose dependent in response to artemisinin (Yan et al., 2015). Such reduction of chlorophyll content can be an appropriate indicator to monitor oxidative damage to plants (Guidi et al., 1997). In the present study, decrease in chlorophyll level of weed plants may be attributed to excessive production of ROS caused by the bioactive compounds present in extracts. *In vivo* investigation of 20% extracts against weeds suggested an induced oxidative stress in them as exhibited by enzymatic and non-enzymatic assays. Phytotoxicity of many allelochemicals can largely be characterized by the induction of free radicals, which consequently produce O^–2^ (Yan et al., 2015). These ROS go through various biochemical pathways to generate even more reactive molecules like OH^–^ or HO_2_. All these free radicals thereafter cause membrane and DNA/protein damage as well as lipid peroxidation that eventually result in cell destruction (Cheng and Cheng, 2015). Ding et al. (2007) also affirmed significant increase in CAT, SOD, and H_2_O_2_ concentration in cucumber, in response to cinnamic acid application. Conversely, it has also been reported that some allelochemicals may reduce SOD and POD activity. The phytotoxic allelochemical, secalonic acid F, derived from the fungus *Aspergillus japonicus*, considerably declined antioxidant enzyme activities in several plants (Zeng et al., 2001). Similarly, rice extracts are also known to inhibit SOD and CAT in barnyard grass (Lin et al., 2000). This indicates that allelochemicals can be directly associated with ROS generation, while elevation of oxidative enzymes is a consequence of production of harmful radicals. In an alternate manner, an allelochemical may also block these enzymes, thus making the plants at high risk of damage.

Phytotoxic effects of bio-extracts on recipient plants are mainly a manifestation of an additive effect of allelopathic compounds that act as toxins when applied exogenously, thus inhibiting weed growth (Afzal et al., 2015). These phytotoxins are mostly characterized as phenolics, as the compounds isolated from one of the allelopathic plants, i.e., *Delonix regia*, were generally phenolic compounds including chlorogenic acid, 3,4-dihydroxy cinnamic acid, 4-hydroxybenzoic acid, 3,5-dinitrobenzoic acid, and gallic acid (Chou and Leu, 1992). Thus, total phenolic and flavonoid content assays were performed on 1, 3, and 5% extracts; and both the phenolic and flavonoid contents were higher in higher concentration (5%). However, the highest concentration of total phenolic acids was found in castor followed by artemisia, wheat, and sorghum, whereas total flavonoids were in the highest amount in artemisia followed by castor, wheat, and sorghum. Secondary metabolites like phenols, flavonoids, alkaloids including terpenes, terpenoids, quinones, isothiocyanates, and benzoxazinoids are widely reported allelochemicals. These bioactive compounds, found abundantly in plant extracts, are pertinently responsible for herbicidal activity (Macías et al., 2019). Differentially expressed metabolites and their qualitative and quantitative analyses through profiling are pivotal to gain insights of species-based differences of donor allelopathic plants. The active phytotoxic metabolites in methanolic fractions prepared from castor, artemisia, wheat, and sorghum extracts were identified and subsequently quantified by HPLC and LC-MS by comparing retention time and area under the peak (abundance) with appropriate standards. HPLC analysis of methanolic fraction of crude extracts showed the presence of phenolic compounds—rutin, quercetin, kaempferol, catechin, gallic acid, vanillic acid, ferulic acid, syringic acid, *p*-hydroxy benzoic acid, *p*-coumaric acid, chlorogenic acid, and caffeic in the extracts. Strong allelopathic potential of studied extracts can be linked to a higher contents of phenolics in them and consequent elevated antioxidant potential, as most of these compounds are reported as phytotoxic (El-Gawad et al., 2019; Elshamy et al., 2019). Sytar (2015) demonstrated that high antioxidant potential of *Fagopyrum esculentum* and *Fagopyrum tataricum rotundatum* was correlated with higher levels of chlorogenic acid, *p*-coumaric acid, trans-ferulic acid, *p*-anisic acid, salicylic acid, methoxycinnamic acid, and vanillic acid in them. Batish et al. (2008) thus attributed phenols in rhizosphere of *Ageratum conyzoides* L., namely, gallic, coumaric, protocatechuic, benzoic, sinapic, *p*-hydroxybenzoic, and coumaric acid, and flavonoids like kaempferol and quercetin, to be responsible for growth reduction in chick pea. Caffeic acid, gallic acid, syringic acid, catechol, *p*-coumaric acid, and *p*-hydroxy benzoic acid, from *Calotropis procera* aqueous leaf extract, also disrupted mitotic index and induced chromosomal abnormalities in *Cassia sophera* L. and *Allium sepa* L., thus reducing their growth and biomass (Gulzar et al., 2016). Phenolic acids (gallic acid, protocatechuic acid, *p*-hydroxybenzoic acid, *p*-hydroxybenzaldehyde, vanillic acid, syringic acid, *p*-coumaric acid, and ferulic acid) and flavonoids (rutin, luteolin, apigenin, and catechin) from *Acacia melanoxylon* R. Br. aqueous extract significantly reduced protein content of *Lactuca sativa* (Hussain et al., 2020). Other phytotoxic effects of phenolics include inhibition of nutrient uptake, respiration, photosynthesis, and enzymatic activities of receiver plants (Li et al., 2010). These investigations verify the correlation between the allelopathic activity of donor plants with their antioxidant potential and their phenol content.

Detailed profiling of allelochemical derivates identified from HPLC was done through MS analysis. Major compounds were derivates of alkaloids, terpenoids, phenolic acids, and flavonoids. Most abundant alkaloids annotated in this study were quinoline, isoquinolines, pyrrolidines, pyridine, terpenoids, and steroids. Sampaio et al. (2018) evaluated the herbicidal activity of alkaloids isolated from *Ruta graveolens* and reported a significant inhibitory effect of 2-(benzo[*d*][1,3]dioxol-5-yl)-1-methylquinolin-4(1*H*)-one (graveolin) on electron transport chain and consequently photosynthetic activity of *Lolium perenne* plants. Apart from having medicinally attributes (Sánchez-Calvo et al., 2016), 1,4-naphthoquinone and sorgoleone are widely studied compounds, containing quinoline rings, with completely analyzed phytotoxicity pathway (Dayan et al., 2010). Quinines induce full plant growth inhibition (Elavarasan and Gopalakrishnan, 2014) due to their immense antioxidant potential (Gan et al., 2017). Propanamide (phenolic acid) derivates detected in our extracts are also a known herbicide for post-emergence inhibition of photosynthesis in monocot and dicot weeds in rice (Kanawi et al., 2016). Our plant fractions were also found to be rich in benzamide and pyrimidine derivatives. Both these classes of compounds exhibit pharmacological properties as antimicrobial, analgesic, anti-inflammatory, anticancer, cardiovascular, antitumor, tuberculostatic, antidiarrhea, anticonvulsants, antibacterial, antimicrobial, and tyrosine kinase inhibitor (Asif, 2013, 2017). Benzamide derivative (4 and 5-chloro-2-hydroxy-*N*-[2-(arylamino)-1-alkyl-2-oxoethyl]benzamides) inhibited the photosynthetic process in spinach, thus demonstrating its herbicidal potential (Imramovsky et al., 2011). Pyrimidine herbicides are also utilized for selective and non-selective weed management in crops (Asif, 2017). Detection of a wide range of unexplored compounds provides a broad spectrum for development of novel herbicides with varied modes of action.

The phytotoxic efficacy of allelopathic bioactive compounds on the target weeds is evident from the results of our studies, proving the natural weed control potential of castor, artemisia, wheat, and sorghum. A combination therapy for weed management may be a step forward by utilizing these allelopathic extracts.

## Conclusion

Castor (*Ricinus communis*), artemisia (*Artemisia santolinifolia*), wheat (*Triticum aestivum*), and sorghum (*Sorghum bicolor*) have immense allelopathic potential against *Sinapis arvensis*, *Lolium multiflorum*, and *Parthenium hysterophorus* weeds. Phytotoxic responses of these bio-extracts were dose dependent. Increase in the herbicidal activity of the extracts with increased concentration can be attributed to higher amount of allelochemicals. Presence of putative allelopathic compounds in these plant fractions makes them an effective source of a natural bio-herbicide formulation.

The plants investigated in this study for allelopathic potential are already largely distributed and cultivated for food, feed, medicinal, and industrial purposes; thus, their large-scale cultivation is already in process. Therefore, these plants as a whole and/or their parts can be utilized for extraction of bioactive compounds having bioherbicidal activity. This may be cost-effective and sustainable production, without disturbing the ecosystem or biodiversity. Further, assessment of allelopathicity of identified derivates of compounds may provide a foundation platform for development of eco-friendly analogs for efficient weed management practices.

## Data Availability Statement

All datasets generated for this study are included in the article/Supplementary Material, further inquiries can be directed to the corresponding author/s.

## Author Contributions

SA performed the experimental procedures and wrote the manuscript. SN helped in experiments planning and execution and reviewed the manuscript. SK helped in experimentation. MA helped in HPLC and LC-MS analyses and reviewed the manuscript. AA reviewed the manuscript. ZA conceptualized, supervised, and executed the research project. All the authors have read and approved the final manuscript.

## Conflict of Interest

The authors declare that the research was conducted in the absence of any commercial or financial relationships that could be construed as a potential conflict of interest.

## Publisher’s Note

All claims expressed in this article are solely those of the authors and do not necessarily represent those of their affiliated organizations, or those of the publisher, the editors and the reviewers. Any product that may be evaluated in this article, or claim that may be made by its manufacturer, is not guaranteed or endorsed by the publisher.
