# Supplementary material for: Bioherbicidal Activity and Metabolic Profiling of Potent Allelopathic Plant Fractions Against Major Weeds of Wheat—Way Forward to Lower the Risk of Synthetic Herbicides
Source: Front Plant Sci. 2021 Sep 10;12:632390. doi: 10.3389/fpls.2021.632390 (PMC8461335; doi:10.3389/fpls.2021.632390)
Supplement: Supplementary file 1 [file Data_Sheet_1.docx]

***Supplementary Material***

1. **Supplementary Figures and Tables**
   1. **Supplementary Figures**


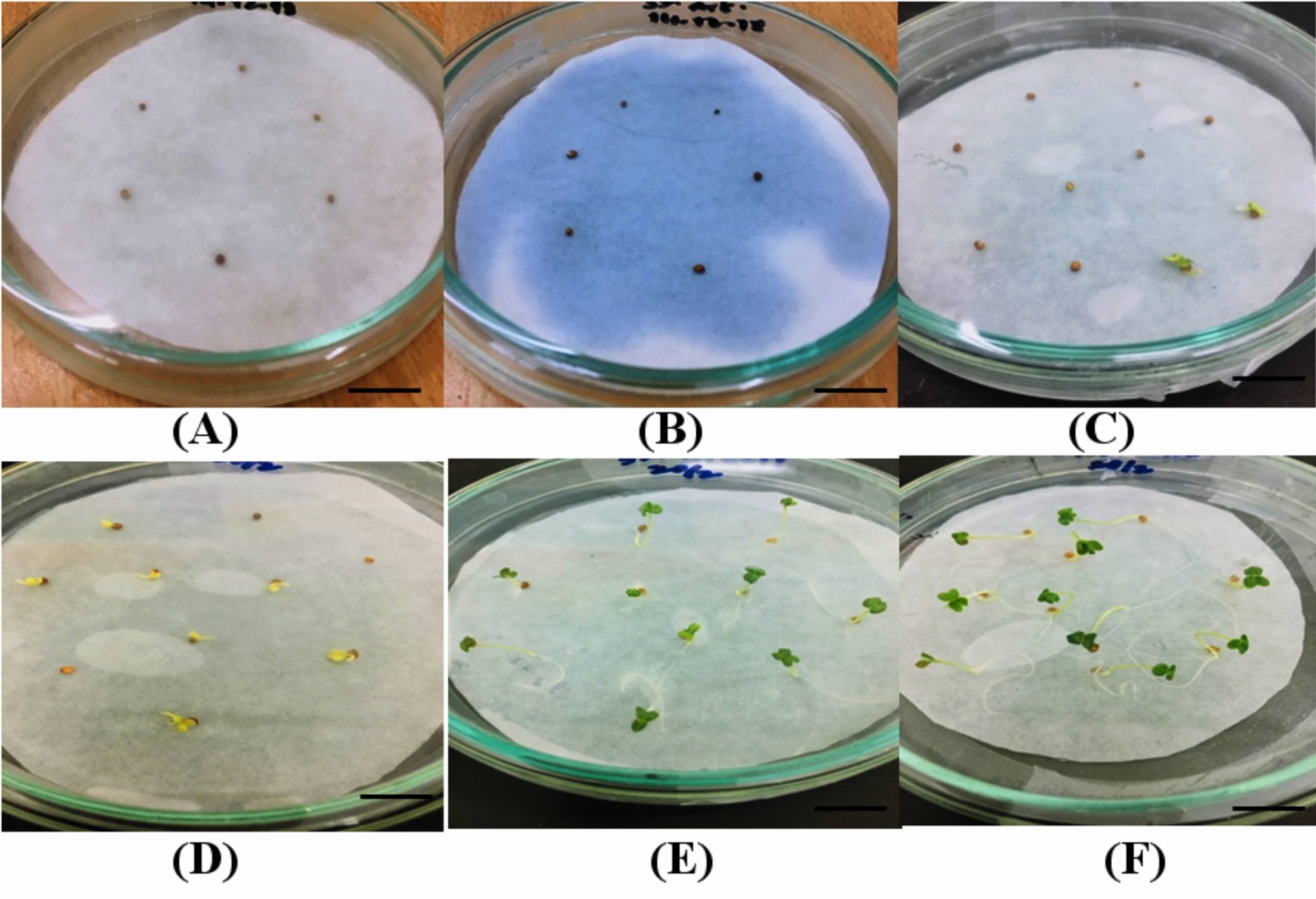


**Supplementary figure 1.** Effect of 5% extracts, solvent and aqueous control on *S. arvensis* seed germination. **(A)** CE **(B)** AE **(C)** SE **(D)** TE **(E)** 5% methanolic control **(F)** aqueous control. CE and AE induced 100% germination inhibition while 80-90% inhibition was caused with SE and TE. Control seeds germinated in time. Data were recorded at 3^rd^ and 5^th^ day of experiment.


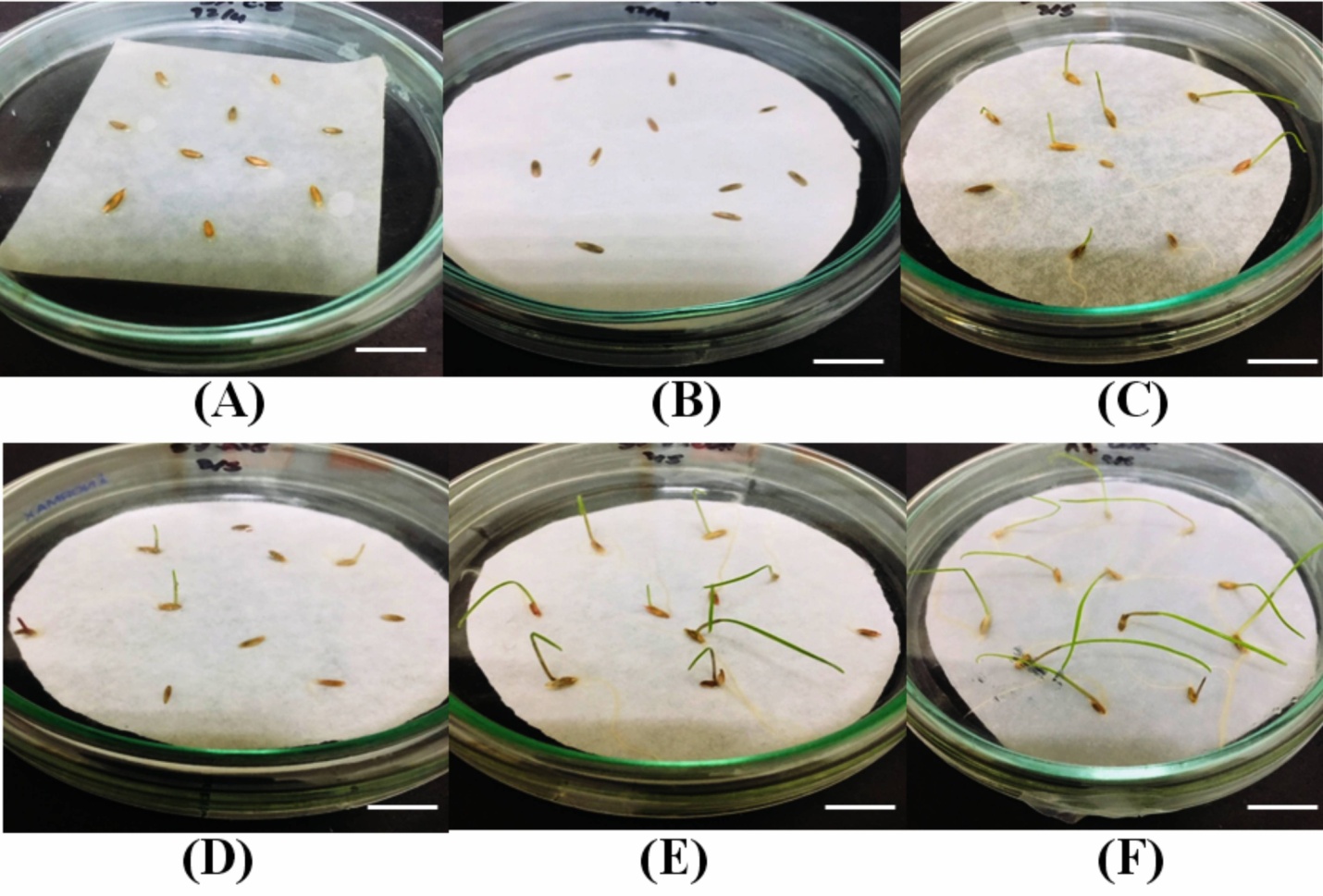


**Supplementary figure 2.** Effect of 5% extracts, solvent and aqueous control on *L. multiflorum* seed germination of **(A)** CE **(B)** AE **(C)** SE **(D)** TE **(E)** 5% methanolic control **(F)** aqueous control. CE and AE induced 100% germination inhibition while 70-90% inhibition was caused with SE and TE. Control seeds germinated in time. Data were recorded at 5^th^ and 10^th^ day of experiment.

**
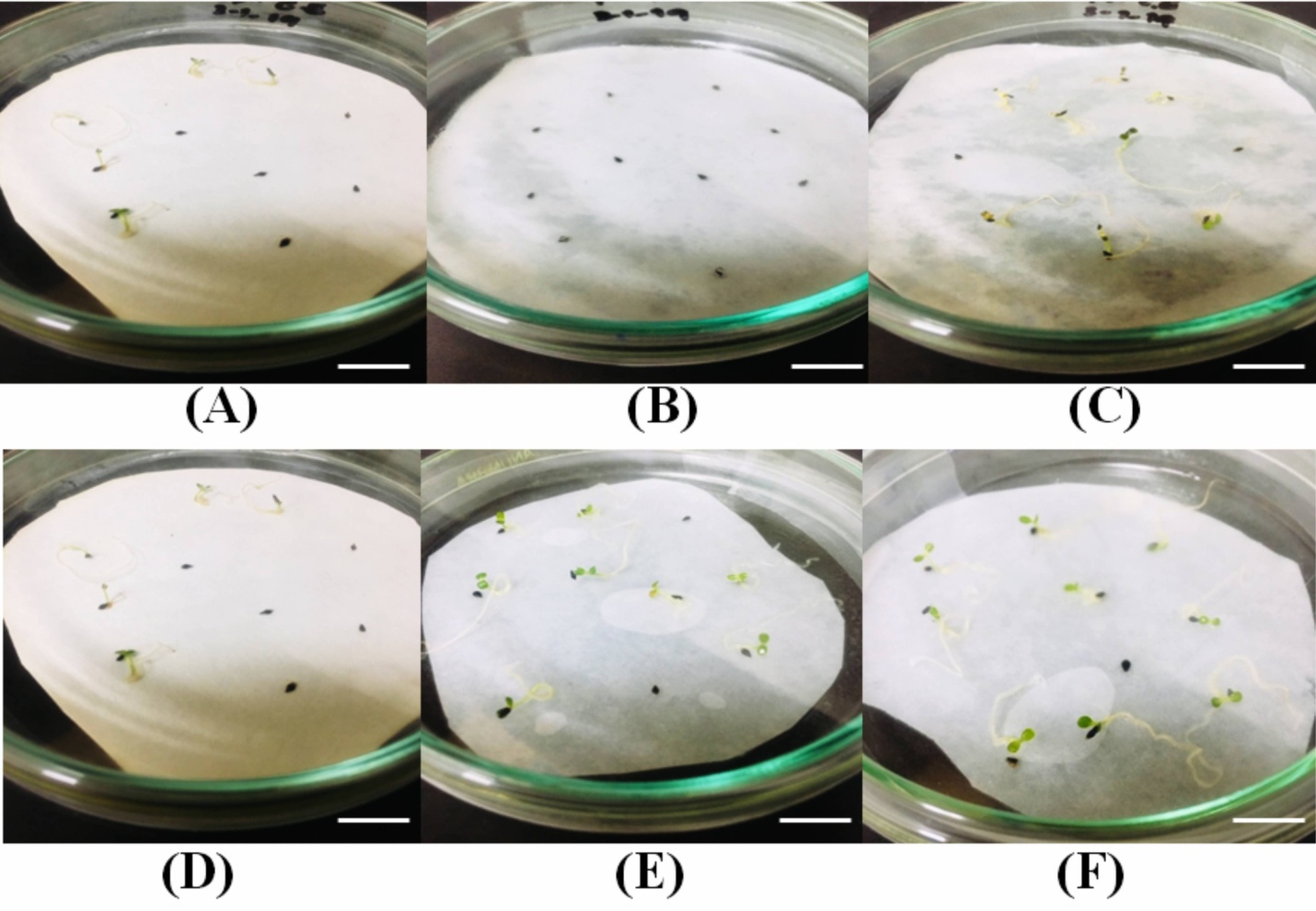
**

**Supplementary figure 3.** Effect of 5% extracts, solvent and aqueous control on *P. hysterophorus* seed germination of **(A)** CE **(B)** AE **(C)** SE **(D)** TE **(E)** 5% methanolic control **(F)** aqueous control. 40-100% germination inhibition was induced with AE, CE, TE and SE respectively. Control seeds germinated in time. Data were recorded at 10^th^ and 15^th^ day of experiment.


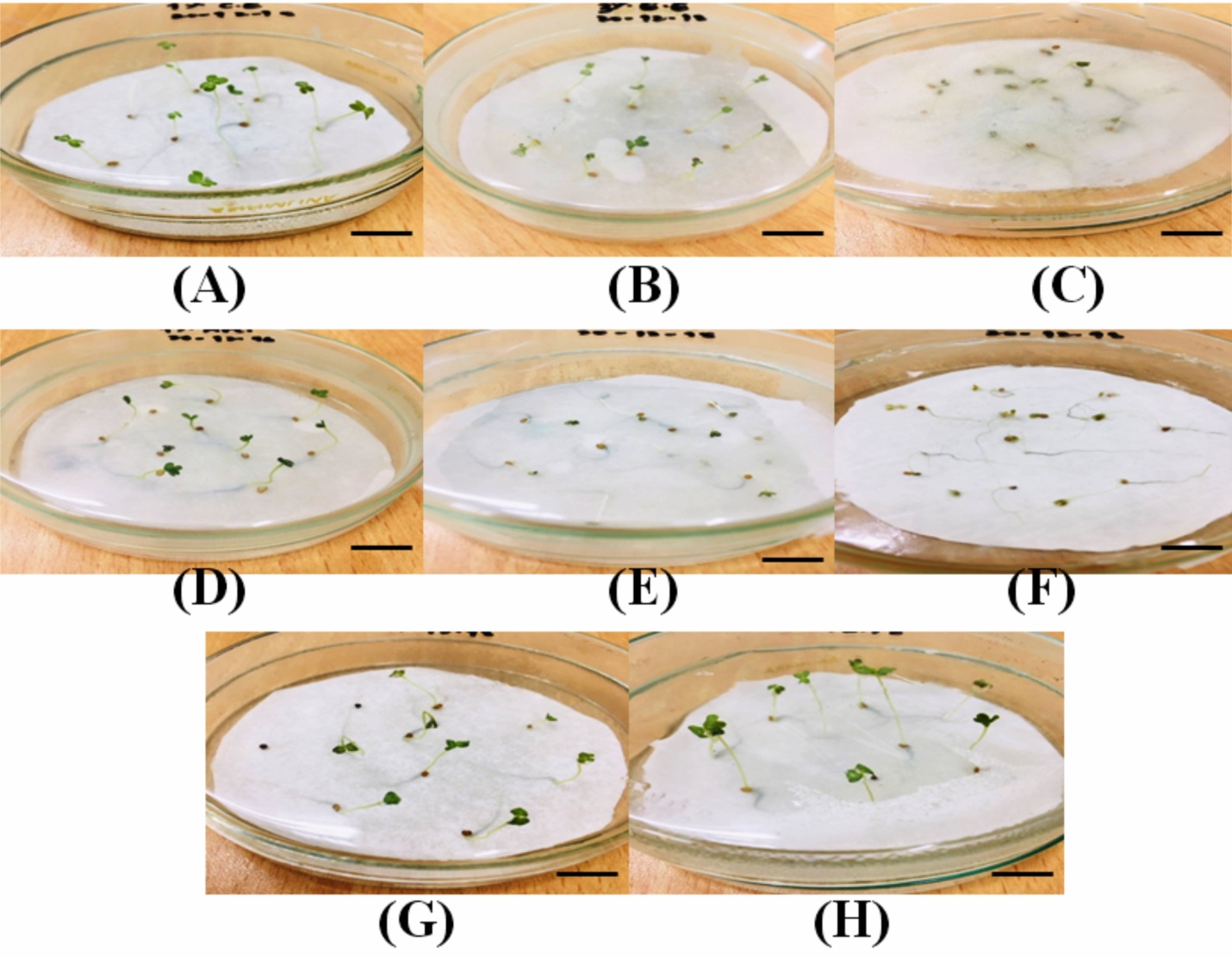


**Supplementary figure 4.** Effect of extracts, solvent and aqueous control on seedling growth of *S. arvensis* after treatments. **(A)** 1% CE **(B)** 3% CE **(C)** 5% CE **(D)** 1% AE **(E)** 3% AE **(F)** 5% AE **(G)** 5% methanol control **(H)** aqueous control. 1% CE did not show significant seedling growth inhibition, 3% CE and AE stunted the growth while 5% of both extracts completely wilted seedling growth. All seedlings grew well with methanol and aqueous control treatments.


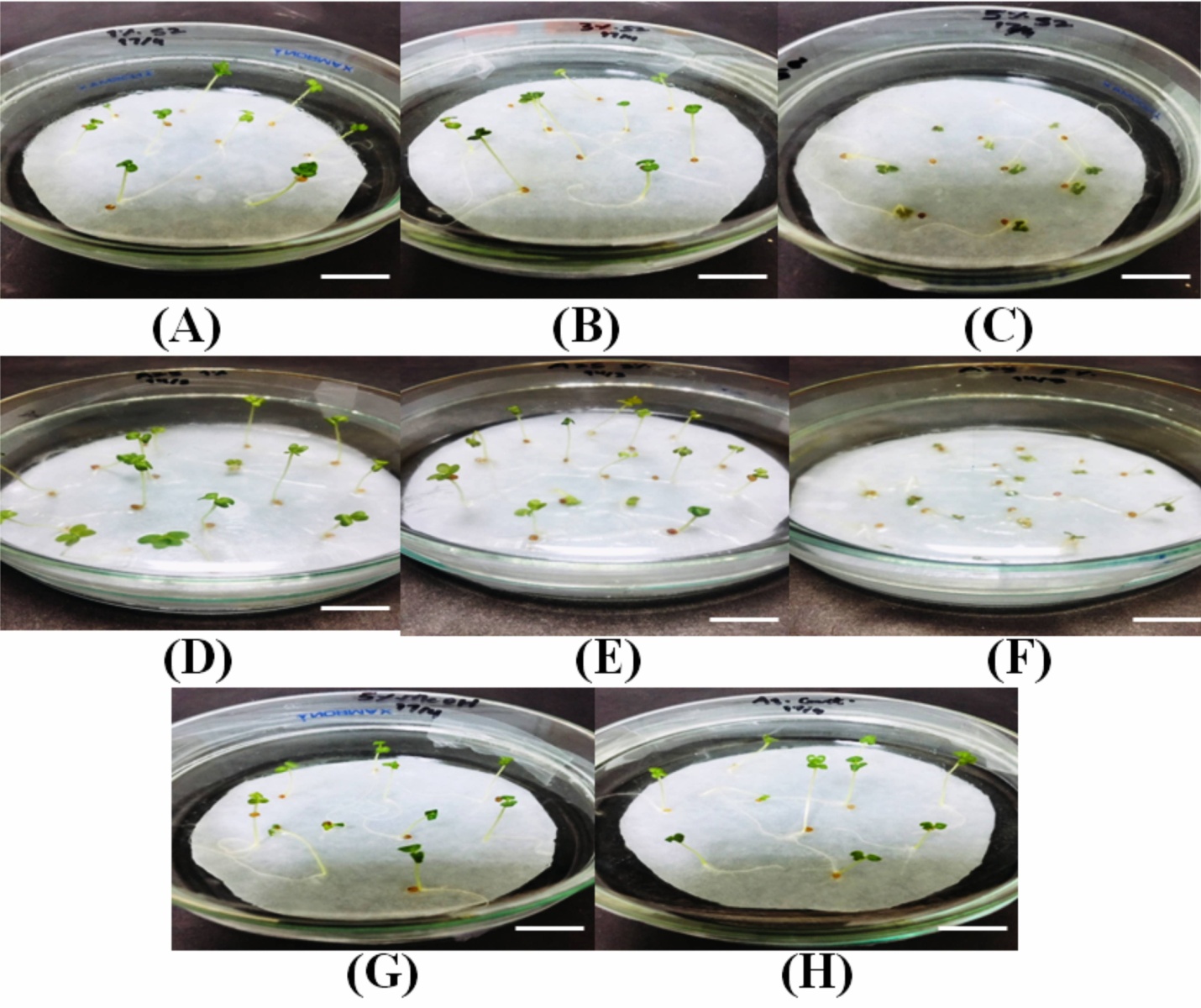


**Supplementary figure 5.** Effect of extracts, solvent and aqueous control on seedling growth of *S. arvensis* after treatments. **(A)** 1% SE **(B)** 3% SE **(C)** 5% SE **(D)** 1% TE **(E)** 3% TE **(F)** 5% TE **(G)** 5% methanol control **(H)** aqueous control. 1% and 3% SE did not show significant seedling growth inhibition, 3% SE and TE stunted the growth while 5% of both extracts completely wilted seedling growth. All seedlings grew well with methanol and aqueous control treatments.


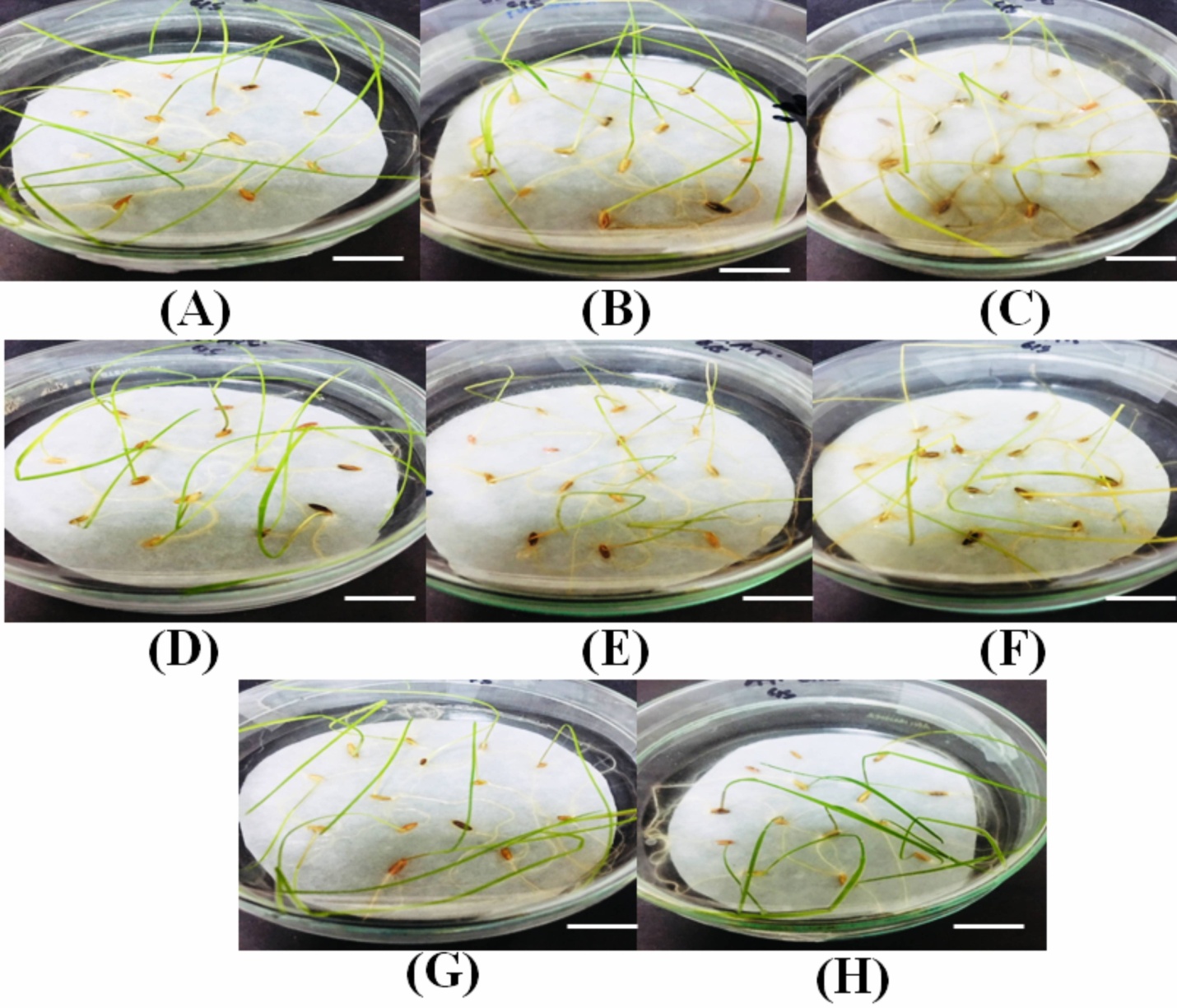


**Supplementary figure 6.** Effect of extracts, solvent and aqueous control on seedling growth of *L. multiflorum* after treatments with **(A)** 1% CE **(B)** 3% CE **(C)** 5% CE **(D)** 1% AE **(E)** 3% AE **(F)** 5% AE **(G)** 5% methanol control and **(H)** aqueous control. Stunted growth was observed with 1 and 3% of CE and AE while 5% of both extracts wilted and bleached the seedlings, showing decrease in amount of chlorophyll. Control treatments did not have any effect on seedling growth.


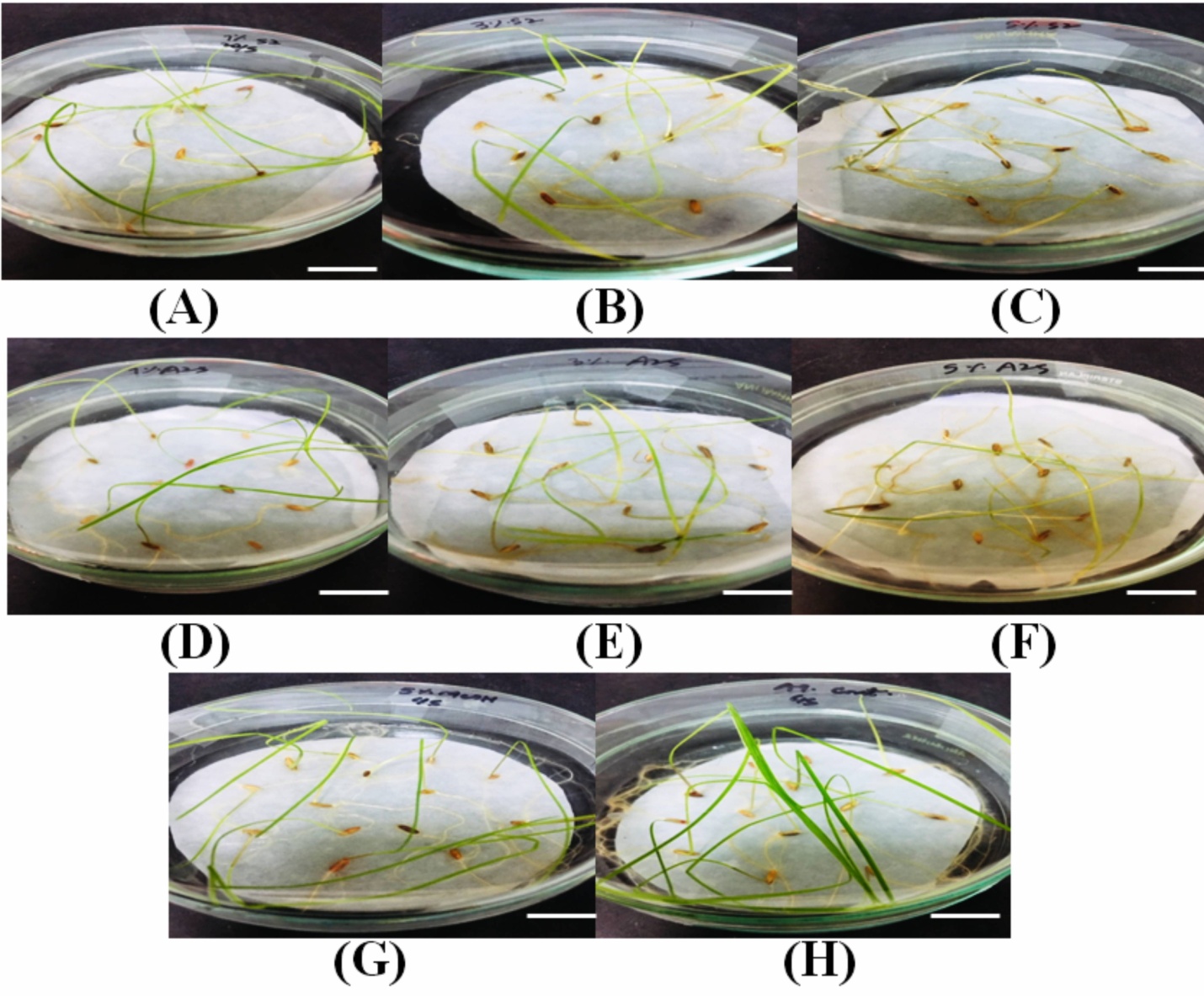


**Supplementary figure 7.** Effect of extracts, solvent and aqueous control on seedling growth of *L. multiflorum* after treatments with **(A)** 1% SE **(B)** 3% SE **(C)** 5% SE **(D)** 1% TE **(E)** 3% TE **(F)** 5% TE **(G)** 5% methanol control and **(H)** aqueous control. Stunted growth was observed with 3% SE and TE while 5% of both extracts produced bleaching effect in seedlings, showing decrease in amount of chlorophyll. Control treatments did not have any effect on seedling growth.


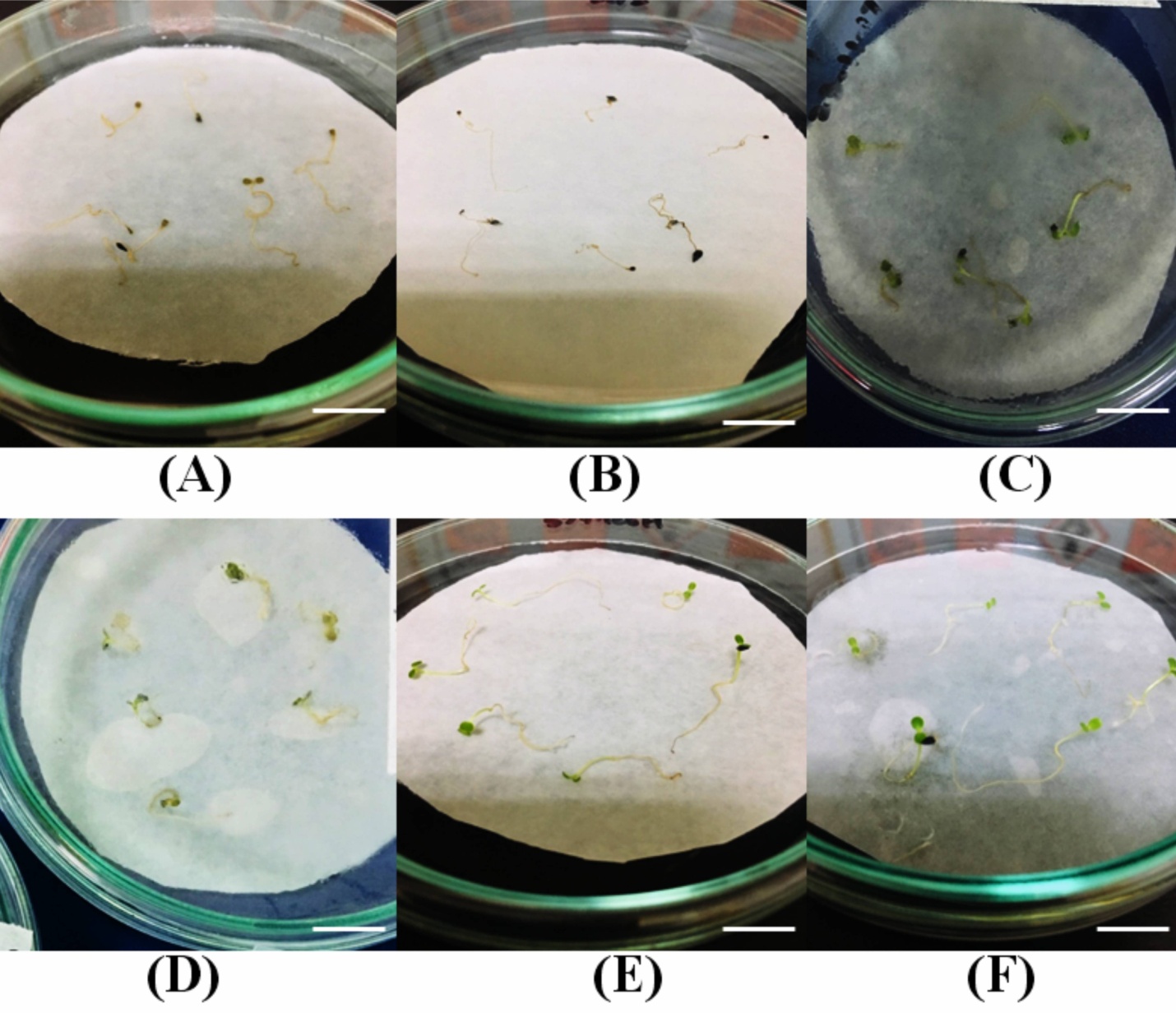


**Supplementary figure 8.** Effect of extracts, solvent and aqueous control on seedling growth of *P. hysterophorus* after treatments with **(A)** 5% CE **(B)** 5% AE **(C)** 5% SE **(D)** 5% TE **(E)** 5% methanol control and **(F)** aqueous control. Stunted growth was observed with all the extracts showing dried up seedlings. Control treatments did not have any effect on seedling growth.


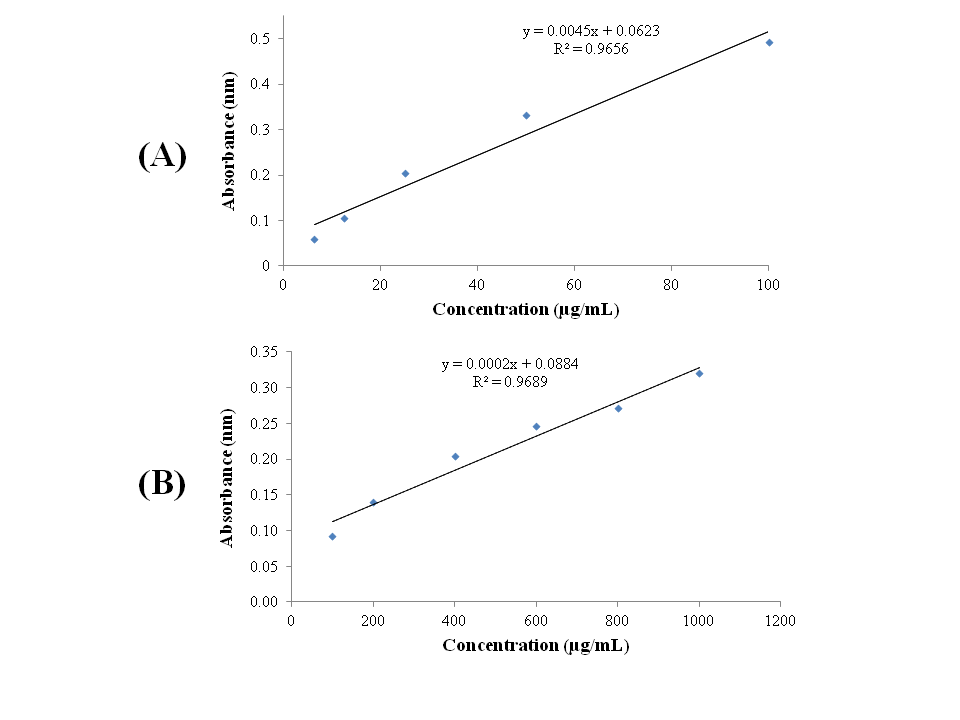


**Supplementary figure 9.**  **(A)** Standard curve of gallic acid for total phenolic content measurement **(B)** Standard curve of quercetin for total flavonoid content measurement.


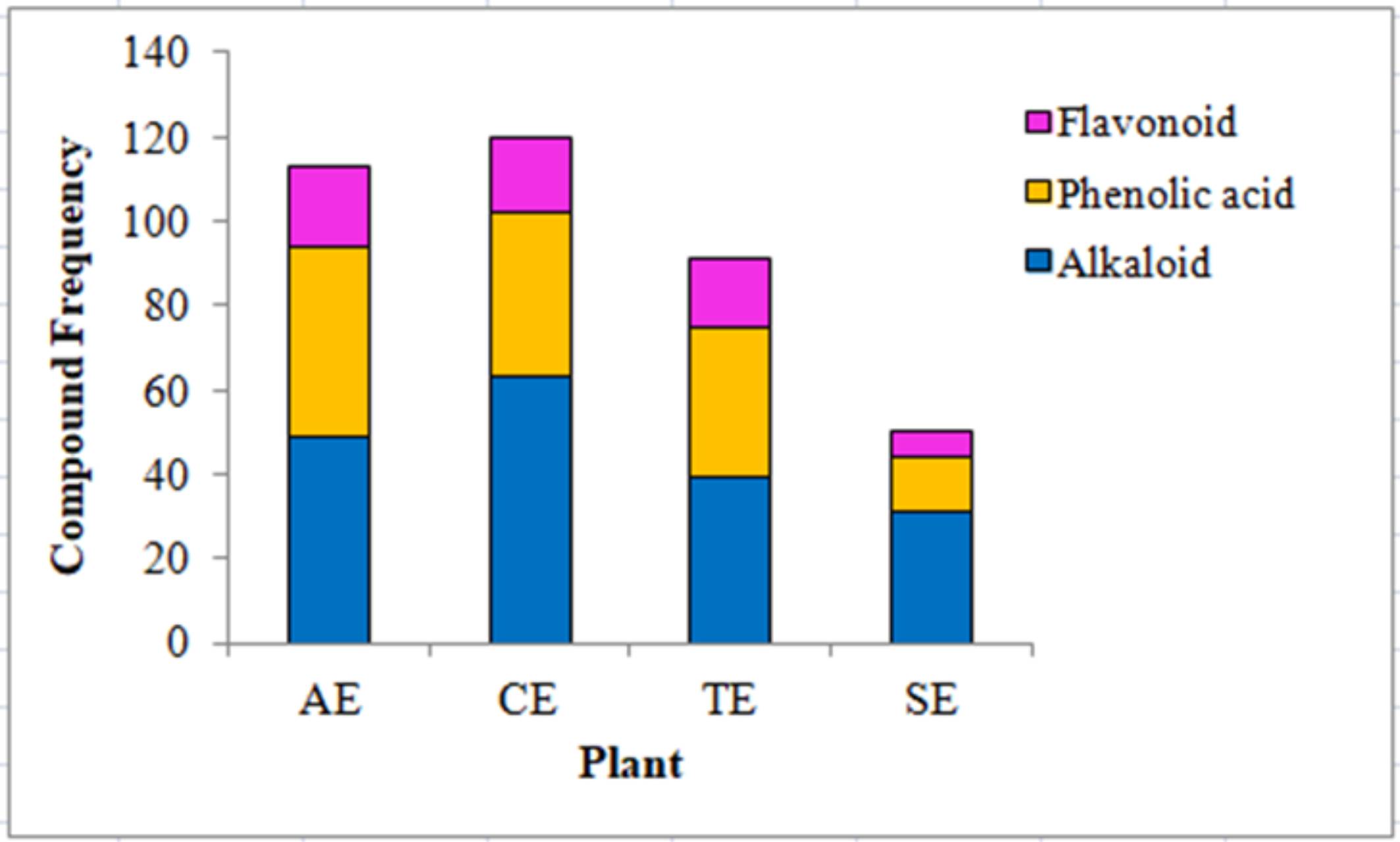


**Supplementary figure 10.**  Frequency of alkaloids, phenolic acids and flavonoids identified through LC-MS in each of AE, CE, TE and SE respectively.

- 1. **Supplementary Tables**

**Supplementary table 1.** Compounds identified by LC- MS in CE.

| Compound  identified | Molecular  formula | RT (min.) | Area % | Match  score | Compound  class |
| --- | --- | --- | --- | --- | --- |
| Thiosulfuric acid S-2-[[2-4-methyl-2-quinoyl]oxy]ethyl]amino]ethyl ester | C_14_H_18_N_2_O_4_S_2_ | 0.9939 | 1.32 | 42.5 | alkaloid |
| Purine-2,6-dione, 1,3-dimethyl-7-(2-oxo-2- phenylethyl)-8-(piperidin-1-yl)-3,7-dihydro- | C_20_H_23_N_5_O | 1.2054 | 3.57 | 50.0 | alkaloid |
| Decahydronaphtho[2,3-b]furan-2-one, 3-pyrrol[2-(4-fluorophenyl)ethylamino]methylmorpho-8a-methyl-5- methylene- | C_23_H_30_FNO_2_ | 1.6602 | 2.53 | 48.2 | alkaloid |
| Benzo[a]phenanthridin-4(3H)-one, 1,2,5,6-tetrahydro- 2,2-dimethyl-5-(3-trifluoromethylphenyl)- | C_26_H_22_F_3_NO | 2.2949 | 2.44 | 51.1 | alkaloid |
| Tetraethyl 1,1'-(1,8-naphthylene)bis(1,2,3-triazole- 4,5-dicarboxylate) | C_26_H_26_N_6_O_8_ | 3.0035 | 0.09 | 42.3 | alkaloid |
| Propanamide, 2-amino-3-phenyl | C_9_H_12_N_2_O | 3.7651 | 23.74 | 74.6 | phenolic acid |
| Isonipecotic acid, N-(bromoacetyl)-, decyl ester | C_18_H_32_BrNO_3_ | 4.6536 | 0.13 | 47.5 | alkaloid |
| Quinine acetate | C_22_H_26_N_2_O_3_ | 4.7382 | 0.18 | 43.2 | alkaloid |
| 6-Hydroxy-3'-methoxyflavone | C_16_H_12_O_4_ | 4.7382 | 1.85 | 55.0 | flavonoid |
| 2(1H)-Pyridinone, 1-cyclohexyl-3,4,5,6-tetramethyl- | C_15_H_23_NO | 6.9912 | 2.59 | 51.9 | alkaloid |
| 6,7-Epoxypregn-4-ene-9,11,18-triol-3,20-dione, 11,18-diacetate | C_25_H_32_O_8_ | 7.0546 | 1.62 | 50.1 | flavonoid |
| Fumaric acid, monoamide, N-methyl-N-phenyl-, 4- chloro-2-methylphenyl ester | C_18_H_16_ClNO_3_ | 7.9749 | 6.90 | 62.9 | phenolic acid |
| 1,3-Dimethoxy-5-(1-methyl-heptyl)-benzene | C_16_H_26_O_2_ | 8.5672 | 0.09 | 34.9 | phenolic acid |
| Isoquinoline, 1,2,3,4-tetrahydro-1-(4-bromophenyl)- 6,7-dimethoxy- | C_17_H_18_BrNO_2_ | 8.5672 | 0.10 | 38.3 | alkaloid |
| 4-Amino-3-methoxypyrazolo[3,4-d]pyrimidine | C_6_H_7_N_5_O | 9.1278 | 41.69 | 56.9 | alkaloid |
| Benzoic acid, 4-propyl-, octadecyl ester | C_28_H_48_O_2_ | 9.6778 | 2.52 | 38.2 | phenolic acid |
| Quinoline, 6,8-dichloro-4-bromoacetyl-2-(3- acetoxyphenyl)- | C_19_H_12_BrCl_2_NO_3_ | 10.1432 | 0.03 | 37.3 | alkaloid |
| Piperazine, N-(2-furoyl)-N'-[2-(4-chlorophenyl)-3- morpholinopropenothioyl]- | C_22_H_24_ClN_3_O_3_S | 10.5240 | 0.26 | 33.8 | alkaloid |
| 4-Amino-2-(p-tolyl)-5H-(1)benzopyrano(4,3- d)pyrimidin-5-one | C_18_H_13_N_3_O_2_ | 10.8519 | 0.38 | 47.0 | alkaloid |
| 3-(3-Benzyloxy-phenyl)-5-chloro-[1,2,4]triazolo[4,3- a]pyridine | C_19_H_14_ClN_3_O | 11.2961 | 0.34 | 39.7 | alkaloid |
| 2,6-Difluoro-3-methylbenzoic acid, nonadecyl ester | C_27_H_44_F_2_O_2_ | 11.4548 | 0.73 | 34.2 | phenolic acid |
| 2-Chloro-2'-hydroxy-4'-methylbenzophenone, tert- butyldimethylsilyl ether | C_20_H_25_ClO_2_Si | 11.6135 | 0.34 | 30.2 | phenolic acid |
| 5-Isobutyl-2-methyl-furan-3-carboxylic acid naphthalen-1-ylamide | C_20_H_21_NO_2_ | 11.8250 | 0.07 | 32.7 | flavonoid |
| 2-Amino[1,3]thiazolo[4,5-d]pyrimidine-5,7-diol triTBDMS | C_23_H_46_N_4_O_2_SSi_3_ | 11.9837 | 0.14 | 35.7 | alkaloid |
| 3H-Naphtho[2,3-b]furan-2-one, 3-[[2-(4-fluorophenyl) ethylamino]methyl]-5,8a-dimethyl- 3a,5,6,7,8,8a,9,9a-octahydro- | C_23_H_30_FNO_2_ | 12.3750 | 0.66 | 35.0 | flavonoid |
| Pyridine-3-carbonitrile, 1,4-dihydro-2-amino-1-(3-cyano-4-ethyl-5-methyl-2-thienyl)-4,4- bis(trifluoromethyl)-2-methyl- | C_17_H_14_F_6_N_4_S | 12.7241 | 1.28 | 38.3 | alkaloid |
| Androstan-17-one, (5.alpha.,14.beta.)- | C_19_H_30_O | 13.0520 | 0.61 | 30.2 | flavonoid |
| 1H-Indene-1,3(2H)-dione, 2-(9H-xanthene-9-yl)- | C_22_H_14_O_3_ | 13.8770 | 0.68 | 36.6 | flavonoid |
| Cinnamic acid, .alpha.-[N-benzoylamino]-3,5-di-t- butyl-4-hydroxy- | C_25_H_31_NO_4_ | 14.1732 | 0.10 | 33.8 | phenolic acid |
| (4-Methoxy-phenyl)-naphthalen-2-ylmethylene-amine | C_18_H_15_NO | 14.4270 | 0.02 | 30.9 | flavonoid |
| 7-Methoxy-2-propyl-4-quinolinol, trimethylsilyl ether | C_16_H_23_NO_2_Si | 14.5539 | 0.03 | 31.4 | alkaloid |
| 4-Hydroxymandelic acid, ethyl ester, di-PFP | C_16_H_10_F_10_O_6_ | 14.8501 | 0.99 | 39.6 | phenolic acid |
| 1,3-oxazino[6,5-g][1,3]benzoxazine, 5,10- dihexadecyl-2,3,4,7,8,9-hexahydro-3,8-dimethyl- | C_44_H_80_N_2_O_2_ | 15.1040 | 0.37 | 32.8 | flavonoid |
| 6H-Pyrazolo[3,4-H]quinazoline, 2-amino-9-methyl-7- (pyridin-2-yl)-5,7-dihydro- | C_15_H_14_N_6_ | 15.5694 | 1.28 | 32.6 | alkaloid |
| L-Homophenylalanine, N,N-bis(3-methylbutyl)-, 3- methylbutyl ester | C_25_H_43_NO_2_ | 16.0771 | 0.14 | 36.0 | phenolic acid |
| Dibenzene, 2,2',5,5'-tetrafluoro-4,4'-dihydroxy-3,3'- dimethoxy- | C_14_H_10_F_4_O_4_ | 16.2463 | 0.05 | 33.9 | phenolic acid |
| 4-Methyl-2-(2-nitro-5-piperidin-1-yl-phenyl)-2H- phthalazin-1-one | C_20_H_20_N_4_O_3_ | 16.3415 | 0.14 | 35.6 | alkaloid |

**RT = retention time**

**Supplementary table 2.** Compounds identified by LC- MS in AE.

| Compound  identified | Molecular formula | RT (min.) | Area % | Match score | Compound class |
| --- | --- | --- | --- | --- | --- |
| Propanamide, N-(4-chlorophenyl)- | C_9_H_10_ClNO | 0.9727 | 0.85 | 51.1 | phenolic acid |
| Benzeneacetic acid, 2-phenylethyl ester | C_16_H_16_O_2_ | 1.1419 | 3.95 | 49.5 | phenolic acid |
| Dimethylmalonic acid, 3-phenylpropyl tridecyl ester | C_27_H_44_O_4_ | 1.1948 | 9.74 | 34.1 | phenolic acid |
| 3,11-Diazatricyclo[7.3.1.0(3.8)]trideca-5,7-dien-4-one, 11-(4-hydroxy-2-butynyl)- | C_15_H_18_N_2_O_2_ | 1.6602 | 4.46 | 40.1 | alkaloid |
| Benzo[a]phenanthridin-4(3H)-one, 1,2,5,6-tetrahydro- 2,2-dimethyl-5-(3-hydroxyphenyl)- | C_25_H_23_NO_2_ | 2.2631 | 2.38 | 49.0 | alkaloid |
| Propanamide, 2-amino-3-phenyl | C_9_H_12_N_2_O | 3.7968 | 5.00 | 59.5 | phenolic acid |
| Gibberellic acid | C_19_H_22_O_6_ | 4.4844 | 0.13 | 44.1 | phenolic acid |
| Quinine acetate | C_22_H_26_N_2_O_3_ | 4.6853 | 0.20 | 41.7 | alkaloid |
| 3,9-Epoxypregn-16-en-14-ol-20-one, 11,18-diacetoxy- 3-methoxy- | C_26_H_36_O_8_ | 4.7700 | 0.17 | 50.0 | flavonoid |
| 3,9-Epoxypregn-16-ene-14-18-diol-20-one, 7,11- diacetoxy-3-methoxy- | C_26_H_36_O_9_ | 6.3142 | 0.41 | 51.0 | flavonoid |
| Acetamide, N-[1,2,3,4-tetrahydro-1-(2-furoyl)-2- methyl-4-quinolinyl]-N-phenyl- | C_23_H_22_N_2_O_3_ | 8.0383 | 2.91 | 49.0 | alkaloid |
| (22R)-6.alpha.,11.beta.,21-Trihydroxy-16.alpha.,17.alpha.-propylmethylenedioxypregna-1,4-diene-3,diene-3,20-dione | C_25_H_34_O_7_ | 9.0009 | 0.25 | 43.6 | flavonoid |
| 2-Formylfuro[2,3-b]pyridin-3-yl acetate | C_10_H_7_NO_4_ | 9.4874 | 5.58 | 41.0 | alkaloid |
| Butane, 1,4-bis(9,10-dihydro-9-methylanthracen-10- yl)- | C_34_H_34_ | 9.8788 | 2.28 | 40.4 | phenolic acid |
| 1,3-Benzodioxole-5-carboxylic acid, 6-acetamino- | C_10_H_9_NO_5_ | 10.1221 | 1.75 | 37.2 | flavonoid |
| Cyclopropanecarboxamide, N-[4-(3-methyl-1H- pyrazol-1-yl)phenyl]- | C_14_H_15_N_3_O | 10.5452 | 8.16 | 58.6 | alkaloid |
| Cyclopropanecarboxylic acid, 3-(2,2-dichloroethenyl)- 2,2-dimethyl-, (3-phenoxyphenyl)methyl ester, cis- | C_21_H_20_Cl_2_O_3_ | 11.1057 | 1.31 | 30.9 | phenolic acid |
| 2,4,6-Trimethoxybenzonitrile | C_10_H_11_NO_3_ | 11.2644 | 22.78 | 51.6 | phenolic acid |
| Isoquinoline, 1,2,3,4-tetrahydro-1-(3-fluorophenyl)- 6,7-dimethoxy- | C_17_H_18_FNO_2_ | 12.2375 | 5.68 | 38.8 | alkaloid |
| Mecloqualone | C_15_H_11_ClN_2_O | 12.5125 | 1.00 | 35.6 | alkaloid |
| Methanone, (2-amino-6,7-dihydro-5H-pyrrolo[1,2- a]imidazol-3-yl)(3,4-dimethoxyphenyl)- | C_15_H_17_N_3_O_3_ | 12.6289 | 1.78 | 38.2 | alkaloid |
| Benzoic acid, 3,4-dimethoxy-, 4-[ethyl[2-(4- methoxyphenyl)-1-methylethyl]amino]butyl ester | C_25_H_35_NO_5_ | 12.7241 | 3.15 | 40.3 | phenolic acid |
| 4-(4-Chloro-phenyl)-1-(tetrahydro-furan-2-ylmethyl) 1,4-dihydro-pyridine-3,5-dicarboxylic acid dimethyl ester | C_20_H_22_ClNO_5_ | 13.1366 | 2.91 | 38.8 | alkaloid |
| Pregna-5,7-dien-3-ol, 20-(3-butynyl)- | C_25_H_36_O | 13.4645 | 0.05 | 34.9 | flavonoid |
| Lanostan-12-one | C_30_H_52_O | 13.7183 | 3.56 | 49.0 | flavonoid |
| {4-Methoxy-2- [(trimethylsilyl)oxy]phenyl}(phenyl)methanone | C_17_H_20_O_3_Si | 14.152 | 1.43 | 38.2 | phenolic acid |
| Carbamic acid, 2-iodo-4-methylphenyl-, methyl ester | C_9_H_10_INO_2_ | 14.2366 | 1.77 | 37.4 | phenolic acid |
| 2H,6H-Pyrido[2,1-b]-1,3-thiazine-7-carboxylic acid,3,4,7,8-tetrahydro-9-cyano-6-oxo-8-(2-thienyl)-,methyl ester | C_15_H_14_N_2_O_3_S_2_ | 14.5539 | 0.69 | 36.7 | alkaloid |
| 3,4-Di(pentafluoropropionyloxy) phenylacetic acid, pentafluoropropionic acid anhydride | C_17_H_5_F_15_O_7_ | 14.8395 | 0.35 | 33.6 | phenolic acid |
| 5,5'-Bis[trifluoromethyl]-2,2'-dimethylbiphenyl | C_16_H_12_F_6_ | 15.178 | 0.06 | 31.9 | flavonoid |
| L-Homophenylalanine, N,N-bis(3-methylbutyl)-, 3- methylbutyl ester | C_25_H_43_NO_2_ | 15.8761 | 2.28 | 35.9 | phenolic acid |
| Thiophene-3-carbonitrile, 4-amino-5-(4- fluorobenzoyl)-2-methylamino- | C_13_H_10_FN_3_OS | 15.9925 | 2.92 | 33.0 | alkaloid |
| Naphtho[2,3-b]furan-4,9-dione, 3-(thiophene-2- carbonyl)-2-trifluoromethyl- | C_18_H_7_F_3_O_4_S | 16.5425 | 0.05 | 32.5 | flavonoid |

**RT = retention time**

**Supplementary table 3.** Compounds identified by LC- MS in TE.

| Compound  identified | Molecular  formula | RT (min.) | Area % | Match  score | Compound class |
| --- | --- | --- | --- | --- | --- |
| Thiosulfuric acid S-2-[[2-[[4-methyl-2- quinolyl]oxy]ethyl]amino]ethyl ester | C_14_H_18_N_2O_4S_2_ | 0.9727 | 1.04 | 46.3 | alkaloid |
| Acetic acid, 2-phenylethyl ester | C_10_H_12_O_2_ | 1.1419 | 1.29 | 38.8 | phenolic acid |
| (S)-7-Bromo-3-isobutyl-2-(2-methylbenzyl)-3,4- dihydro-2H-benzo[b][1,4,5]oxathiazepine 1,1-dioxide | C_20_H_24_BrNO_3_S | 1.2054 | 4.39 | 33.1 | alkaloid |
| (S)-7-Bromo-3-isobutyl-2-(2-methylbenzyl)-3,4- dihydro-2H-benzo[b][1,4,5]oxathiazepine 1,1-dioxide | C_20_H_24_BrNO_3_S | 1.2054 | 4.39 | 33.1 | alkaloid |
| Prednisolone | C_21_H_28_O_5_ | 2.5699 | 2.22 | 47.9 | flavonoid |
| Propanamide, 2-amino-3-phenyl | C_19_H_12_N_2_O | 3.7440 | 22.34 | 70.3 | phenolic acid |
| 5,6,7,8-Tetrahydroindolizine | C_8_H_11_N | 3.8497 | 29.59 | 52.4 | alkaloid |
| Fumaric acid, monoamide, N-methyl-N-phenyl-, 4-chloro-2-methylphenyl ester | C_18_H_16_ClNO_3_ | 8.0278 | 22.91 | 69.0 | phenolic acid |
| Benzoimidazol-2-one, 1-adamantan-1-yl-1,3-dihydro- | C_17_H_20_N_2_O | 8.7893 | 0.22 | 40.2 | alkaloid |
| 5H-1,2,3-Dithiazole, 4-chloro-5-[(2-methoxyphenylazo)nitromethylidene]- | C_10_H_7_ClN_4_O_3_S_2_ | 9.2845 | 0.31 | 37.3 | alkaloid |
| 2-Hydroxyethylflurazepam, tert-butyldimethylsilylether | C_23_H_28_ClFN_2_O_2_Si | 9.8867 | 0.62 | 42.0 | alkaloid |
| Thieno[2,3-d]pyrimidine-2-carboxylic acid, 5,6-dimethyl-4-oxo-2-phenyl-1,2,3,4-tetrahydro-, ethyl ester | C_17_H_18_N_2_O_3_S | 10.2173 | 0.08 | 37.4 | alkaloid |
| 2-Chlorophenethyl alcohol, TBDMS derivative | C_14_H_23_ClOSi | 10.6615 | 0.33 | 31.0 | phenolic acid |
| 2-Propenamide, 2-cyano-N,N-dimethyl-3-[4-[[4-(dimethylamino)phenyl]azo]phenyl]- | C_20_H_21_N_5_O | 10.9365 | 1.83 | 40.6 | phenolic acid |
| 7,15-Dihydroxydehydroabietic acid, methyl ester,di(trimethylsilyl)ether | C_27_H_46_O_4_Si2 | 11.0952 | 0.28 | 34.7 | flavonoid |
| Benzoic alcohol, 2-hydroxy-3,5-dinitro- | C_7_H_6_N_2_O_6_ | 11.2538 | 0.72 | 32.3 | phenolic acid |
| 2,6-Difluoro-3-methylbenzoic acid, heptadecyl ester | C_25_H_40_F_2_O_2_ | 11.4760 | 0.97 | 34.1 | phenolic acid |
| 2,3,5,6-Tetrafluorophenol | C_6_H_2_F_4_O | 11.6875 | 0.53 | 32.4 | phenolic acid |
| 7-(3-Methylbutyl)-1H-indole-2,3-dione, TBDMS derivative | C_19_H_29_NO_2_Si | 12.3539 | 0.29 | 32.8 | alkaloid |
| Pyridine-3-carbonitrile, 1,4-dihydro-2-amino-1-(3-cyano-4-ethyl-5-methyl-2-thienyl)-4,4-bis(trifluoromethyl)-2-methyl- | C_17_H_14_F_6_N_4_S | 12.7347 | 0.25 |  | alkaloid |
| N-(2-Fluoro-phenyl)-2-methoxy-4-methylsulfanylbenzamide | C_15_H_14_FNO_2_S | 13.8770 | 1.41 | 34.3 | phenolic acid |
| Hydroxyvalerenic acid, tert.-butyldimethylsilyl ether,tert.-butyldimethylsilyl ester | C_27_H_50_O_3_Si2 | 14.2578 | 0.11 | 30.8 | flavonoid |
| 4-Hexen-3-one oxime, o-[(pentafluorophenyl)methyl]- | C_13_H_12_F_5_NO | 15.4673 | 0.84 | 35.1 | phenolic acid |
| 1(3H)-Isobenzofuranone, 3,3'-(4-methoxy-1,3-phenylene)bis[3-(4-methoxyphenyl)- | C_37_H_28_O_7_ | 16.0771 | 3.04 | 29.3 | flavonoid |

**RT = retention time**

**Supplementary table 4.** Compounds identified by LC- MS in SE.

| Compound  identified | Molecular  formula | RT (min.) | Area % | Match score | Compound class |
| --- | --- | --- | --- | --- | --- |
| Thiosulfuric acid S-2-[[2-[[4methyl-2-quinolyl]oxy]ethyl]amino]ethyl ester | C_14_H_18_N_2_O_4_S_2_ | 0.9883 | 3.03 | 44.5 | alkaloid |
| 1-Methyl-3-phenylpiperazine | C_11_H_16_N_2_ | 1.1737 | 17.20 | 39.9 | alkaloid |
| Quinine | C_20_H_24_N_2_O_2_ | 1.4487 | 5.98 | 43.5 | alkaloid |
| Propanamide, 2-amino-3-phenyl | C_9_H_12_N_2_O | 1.6391 | 45.91 | 66.7 | phenolic acid |
| Quinoxaline, 5-methyl- | C_9_H_8_N_2_ | 9.3499 | 8.85 | 54.0 | alkaloid |
| Isatin biscresol, 3TMS derivative | C_31_H_43_NO_3_Si_3_ | 10.5452 | 1.55 | 38.0 | alkaloid |
| 4-Azatricyclo[5.2.1.0(2,6)]decane-3,5-dione, 4-[4-(2-methylphenoxy)phenyl]- | C_22_H_21_NO_3_ | 10.8836 | 5.25 | 45.3 | alkaloid |
| Benzene, 1-[2-bromo-1-(methoxydiphenylmethyl)ethyl]-4-methoxy- | C_23_H_23_BrO_2_ | 11.5712 | 1.75 | 31.0 | phenolic acid |
| Pyridine-3-carbonitrile, 1,4-dihydro-2-amino-1-(3-cyano-4-ethyl-5-methyl-2-thienyl)-4,4-bis(trifluoromethyl)-2-methyl- | C_17_H_14_F_6_N_4_S | 12.7029 | 1.36 | 35.3 | alkaloid |
| Flumequine, tert-butyldimethylsilyl ester | C_20_H_26_FNO_3_Si | 12.9779 | 1.88 | 31.5 | alkaloid |
| 4-Hydroxymandelic acid, ethyl ester, di-PFP | C_16_H_10_F_10_O_6_ | 14.8501 | 2.25 | 37.2 | phenolic acid |
| 2,4-Dimethoxy-4b,5,6,7,8,8a,9,10-octahydrophenanthren-1-ol-5,10-dione-7-acetic acid,methyl ester | C_19_H_22_O_7_ | 15.9078 | 4.99 | 32.5 | alkaloid |

**RT = retention time**
